# Elevator‐Like Hollow Channels in Porous Scaffolds Accelerate Vascularized Bone Regeneration via NETs‐Fibrin‐Mediated Macrophage Recruitment

**DOI:** 10.1002/advs.202515693

**Published:** 2025-12-05

**Authors:** Guifang Wang, Rongpu Liu, Huijing Ma, Shuhan Duan, Guangzheng Yang, LingXi Meng, Yuqin Qiao, Dongqiang Song, Wenjie Zhang

**Affiliations:** ^1^ Department of Prosthodontics Shanghai Ninth People's Hospital Shanghai Jiao Tong University School of Medicine College of Stomatology Shanghai Jiao Tong University National Center for Stomatology National Clinical Research Center for Oral Diseases Shanghai Key Laboratory of Stomatology Shanghai 200011 China; ^2^ Department of Oral Surgery Shanghai Ninth People's Hospital Shanghai Jiao Tong University School of Medicine College of Stomatology Shanghai Jiao Tong University National Center for Stomatology National Clinical Research Center for Oral Diseases Shanghai Key Laboratory of Stomatology Shanghai 200011 China; ^3^ Materdicine Lab School of Life Sciences Shanghai University Shanghai 200444 China; ^4^ Department of Hepatobiliary Oncology Zhongshan Hospital Fudan University Liver Cancer Institute Zhongshan Hospital Fudan University Shanghai 200032 China

**Keywords:** angiogenic–osteogenic coupling, bone regeneration, neutrophil extracellular traps, silk fibroin scaffold, vascularization

## Abstract

Developing viable tissue‐engineered constructs for large‐scale bone defects remains a fundamental challenge due to the difficulty of establishing adequate vascular networks. Channel structures act as biological elevators, rapidly promoting vascularization in scaffold materials. However, the underlying mechanisms driving this accelerated process remain unclear. In this study, porous silk fibroin (SF) scaffolds with hollow channels are engineered to investigate their vascularization‐accelerating mechanisms. It is demonstrated that the channels enable the rapid infiltration of fibrinogen and platelets. This initiates a sequential biological cascade involving neutrophil recruitment, the formation of neutrophil extracellular traps (NETs), and subsequent macrophage migration. This coordinated process generates a provisional yet bioactive matrix that promotes directional vascular invasion. Leveraging the architecture of the hollow channels, a biomimetic system is developed that rapidly establishes provascular microenvironments featuring macrophage‐populated NETs‐fibrin networks through blood clot preloading. This combined structural and biological strategy enhances angiogenic–osteogenic coupling through spatially controlled bone morphogenetic protein‐2 (BMP‐2) presentation, significantly improving bone regeneration and providing a clinically translatable solution for vascularized bone regeneration. This study not only elucidates the mechanistic link between scaffold architecture and host immune response, but also establishes a novel paradigm for biomaterial design in hard tissue engineering.

## Graphical abstract

This study demonstrates that how hollow‐channel scaffolds promote vascularized bone regeneration via an immunomodulatory mechanism. The channel structures facilitate the formation of a neutrophil extracellular traps‐fibrin scaffold that recruits vascular endothelial growth factor A (VEGF‐A)‐secreting M2 macrophages to drive angiogenesis. Combining this approach with autologous blood clot and localized BMP‐2 release, the strategy significantly enhances angiogenic–osteogenic coupling, improving bone regeneration.

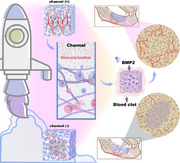

## 1 Introduction

Large bone defects represent a major unmet clinical challenge, with current treatment strategies often failing to achieve complete functional restoration. Reconstructing microvascular networks is essential for successful tissue regeneration and functional recovery.^[^ ^1^, ^2^ ^]^ However, in large‐scale bone tissue engineering, insufficient vascularization, especially in the core region of the critical sized bone tissue constructs, frequently limits regenerative capacity, leading to delayed healing, graft failure, and significant morbidity.^[^ ^3^ ^]^ Accelerating and optimizing vascularization in engineered constructs is therefore a critical challenge in bone tissue engineering.

Over the past three decades, researchers have developed various strategies, primarily involving prevascularization, compositional modification, and structural optimization, to enhance scaffold vascularization. Among these strategies, hollow channel architectures have emerged as a promising approach to enhance vascularization.^[^ ^4^, ^5^, ^6^ ^]^ Channel structures act as biological elevators, providing guided pathways for the rapid infiltration of cells, blood vessels, and tissues. This is a significantly faster alternative to conventional healing processes. Integrating hollow channels into conventional porous scaffolds offers significant advantages in guiding vascularization and directing tissue growth. This approach presents new opportunities for bulk tissue regeneration with substantial clinical potential. Resembling the natural Haversian canal system of native bone,^[^ ^7^, ^8^ ^]^ these bioinspired structures have a precisely controlled spatial structure that minimizes the diffusion distance of oxygen and nutrients from the exterior to the core. They also provide direct, guided pathways for vascular and tissue ingrowth.^[^ ^9^, ^10^, ^11^, ^12^ ^]^ These structures serve as templates for vasculature, guiding the attachment and migration of endothelial cells and promoting anastomosis between the new vasculature and the existing vasculatures, thereby accelerating the formation of vascular networks.

Although hollow channel structures demonstrate empirical benefits in accelerating vascularization, the underlying biological mechanisms remain poorly understood. Elucidating these processes is critical for rational design of optimized scaffolds and addressing vascularization challenges in large bone tissue constructs, offering both fundamental insights and clinical translation potential.

**Figure 1:**
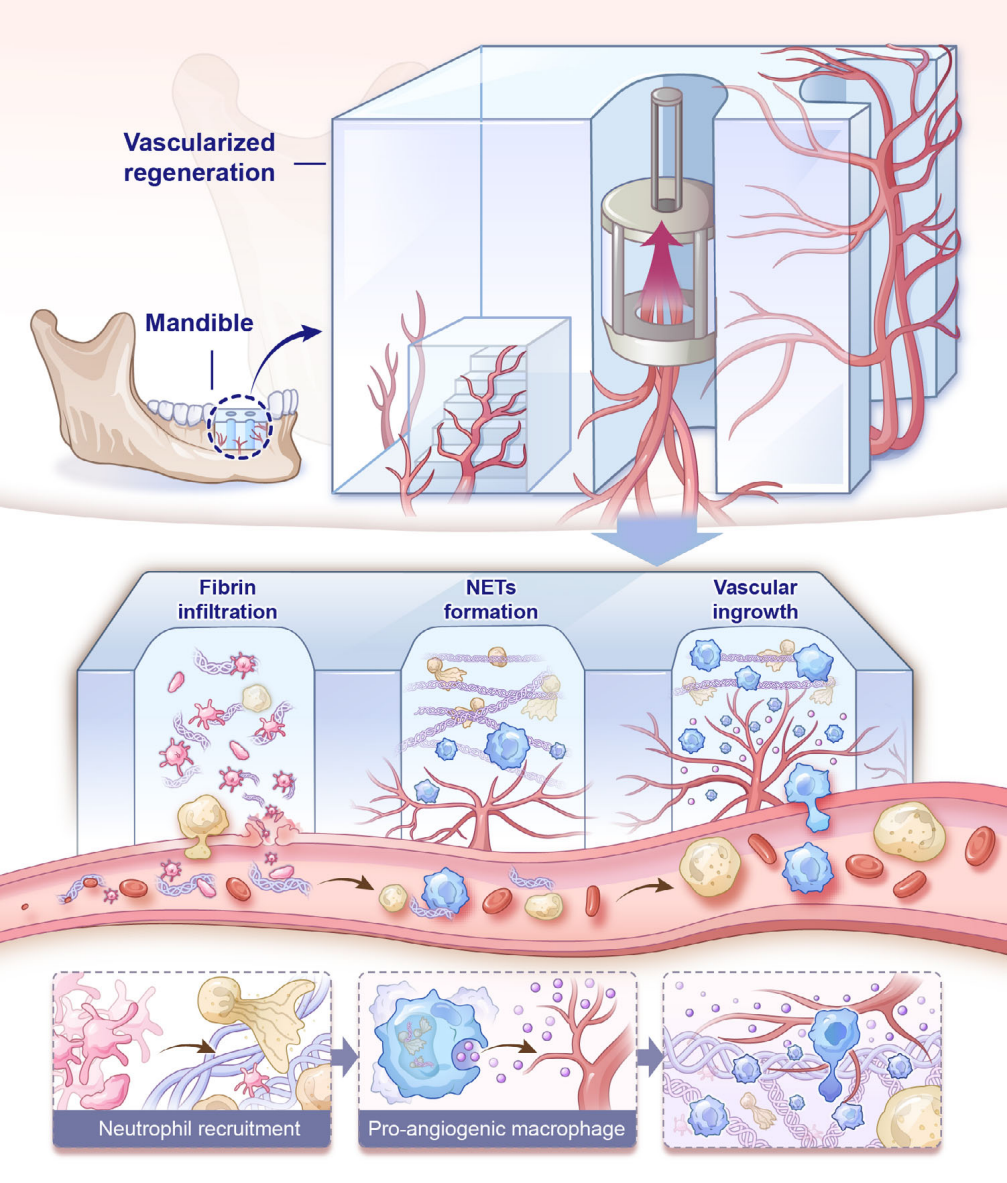
Schematic of the biological process in channel‐mediated vascularization. Channel structure functions as an elevator rapidly driving vascular ingrowth. This architecture enables the rapid infiltration of fibrin and platelets, triggering a sequential cascade of events: neutrophil recruitment, NETs formation, and macrophage migration and activation. This generates a proangiogenic microenvironment that significantly enhances vascularization efficiency.

Leveraging its excellent biocompatibility, tunable degradation into nontoxic products, extracellular matrix‐mimetic three‐dimensional (3D) porous structure, and ability to deliver bioactive components (e.g., VEGF, and BMP‐2),^[^ ^10^, ^13^, ^14^, ^15^ ^]^ we developed porous, channel‐arrayed SF scaffolds—an ideal platform—to investigate the mechanisms underlying hollow channel‐mediated acceleration of vascularization (**Figure** **1**). Through systematic biological analysis, we discovered an unrecognized immune‐mediated mechanism that channel structure orchestrates rapid vascular ingrowth, functioning like a biological elevator system. The channel architectures facilitate the deep and rapid infiltration of fibrin and activated platelets. This infiltration initiates a coordinated cascade involving neutrophil recruitment, NETs formation, and subsequent macrophage migration, generating a proregenerative microenvironment and dramatically boosting vascularization efficiency. Supported the regional distribution of BMP‐2, this method utilizes its capacity to encourage rapid vascular development and augment the angiogenic–osteogenic coupling outcome.

## 2 Results

### 2.1 Hollow Channels Accelerate Vascularized Integration of Implanted Scaffolds In Vivo

**Figure 2:**
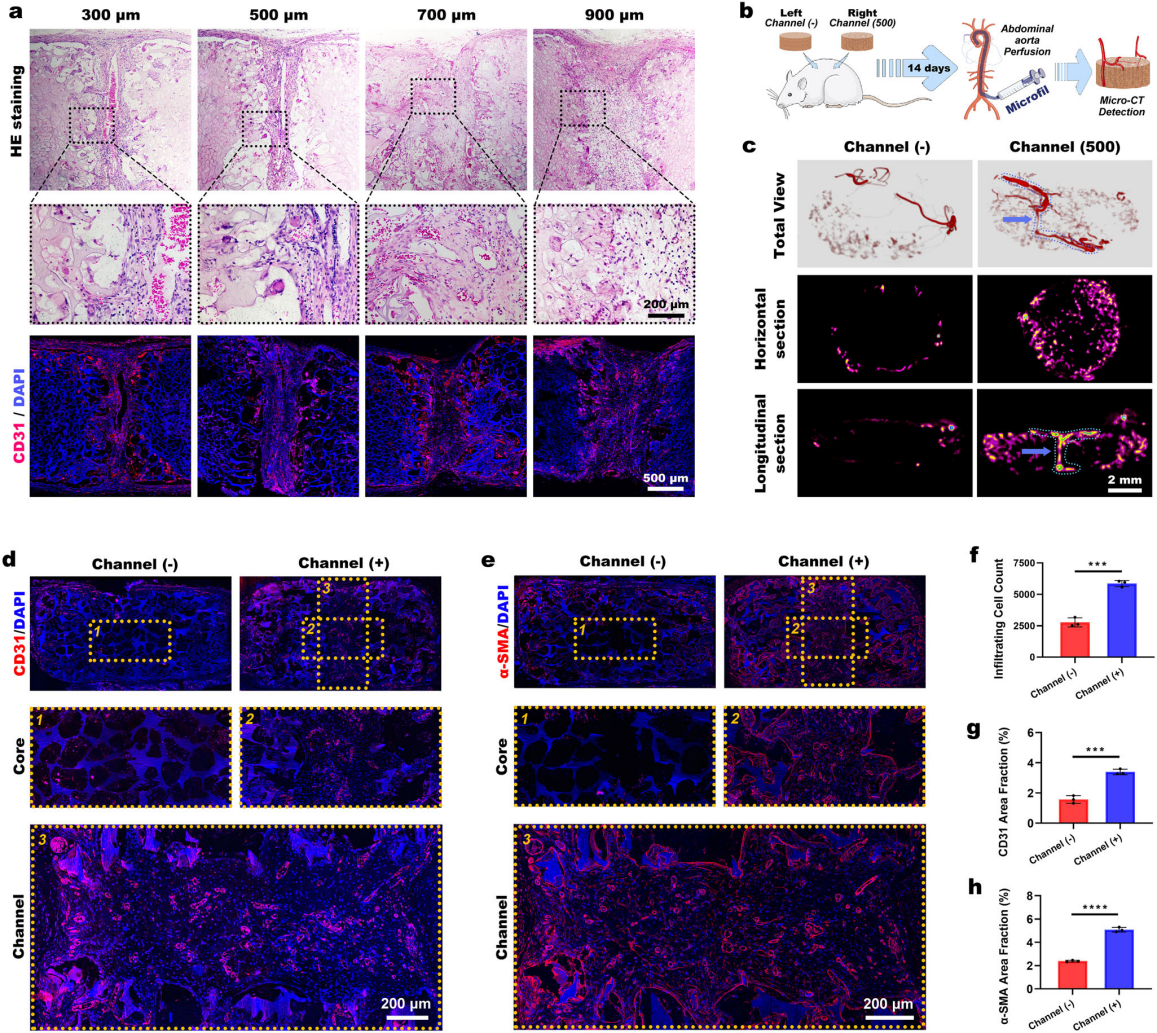
Hollow channel structure promotes scaffolds vascularization. a) H&E staining, and CD31 immunofluorescence staining for vascular ingrowth observation after channeled scaffolds were implanted subcutaneously in rats for 14 days. Nuclei were counterstained by DAPI. b) Experimental workflow showing subcutaneous implantation of channeled/nonchanneled scaffolds in rats, followed by Microfil perfusion via abdominal aorta at day 14 postimplantation. c) Micro‐CT 3D reconstructions of vascular networks in both scaffold types are shown. Orthogonal views are provided: cross‐section perpendicular to channels and longitudinal section parallel to channels. arrow: vascular ingrowth penetrating the scaffold. d) CD31 and e) α‐SMA immunofluorescence staining for vascular ingrowth observation, with nuclei counterstained by DAPI. f–h) The statistical analysis of the total infiltrating cells, CD31 and α‐SMA positive expression areas within the scaffolds is to be conducted (*n* = 3 biological replicates). Data were analyzed by unpaired *t*‐test, ^***^ *p* < 0.001, and ^****^ *p* < 0.0001 indicate statistical significance.

Scaffolds with different channel diameters (300, 500, 700, and 900 µm) were subcutaneously implanted in rats for 2 weeks. The scaffolds were then examined histologically using hematoxylin and eosin (H&E) staining. As shown in **Figure** **2**a, the channel structures consistently supported enhanced cellular and tissue infiltration, regardless of the channel diameter (300, 500, 700, or 900 µm). This enhanced infiltration was also highly vascularized, which was demonstrated by CD31 immunofluorescence staining.

Given that we have previously demonstrated that channels with a diameter of 500 µm could guide rapid tissue ingrowth and support functional vascularization while maintaining scaffold integrity, a channel diameter of 500 µm was selected for subsequent scaffold fabrication (Figure S1, Supporting Information).^[^ ^10^, ^11^ ^]^ Scaffolds with 500‐µm‐diameter hollow channels were subcutaneously implanted in rats for 2 weeks. Microfil perfusion and micro‐CT imaging were performed to evaluate vascularization (Figure 2b). 3D micro‐CT reconstructions (Figure 2c) revealed that the scaffolds without channels exhibited limited vascular encapsulation around the periphery and no significant vascular infiltration into the interior. In contrast, the scaffolds with channels demonstrated extensive vascular networks surrounding and penetrating the scaffolds. Cross‐sectional and longitudinal analyses further highlighted these differences.

For histological observation using H&E and Masson's trichrome staining (Figure S2, Supporting Information) revealed sparse cell infiltration and collagen deposition in the core region of nonchanneled scaffolds. In contrast, channeled scaffolds exhibited robust cellular and collagenous tissue ingrowth throughout the structure, including the core region. These data demonstrated that channeled architectures enhance deep cellular and tissue infiltration compared to porous structures. Immunofluorescence costaining for CD31^+^ endothelial cells and alpha smooth muscle actin (α‐SMA) positive smooth muscle cells demonstrated that nonchanneled scaffolds showed vascular growth restricted to the periphery, with minimal infiltration into the core regions (Figure 2d,e). In contrast, channeled scaffolds exhibited newly formed vessels that permeated the interior, aligned longitudinally, and integrated with the host vasculature. Quantification of infiltrating cells and areas of CD31⁺ and α‐SMA⁺ vasculature (Figure 2f–h) revealed that channeled scaffolds significantly increased cellular infiltration and neovascularization. These results suggest that channels are an effective strategy for improving vascularization and tissue‐scaffold integration.

### 2.2 Immune‐Complex Scaffold Guides Directional Angiogenesis through the Channel Structure

**Figure 3:**
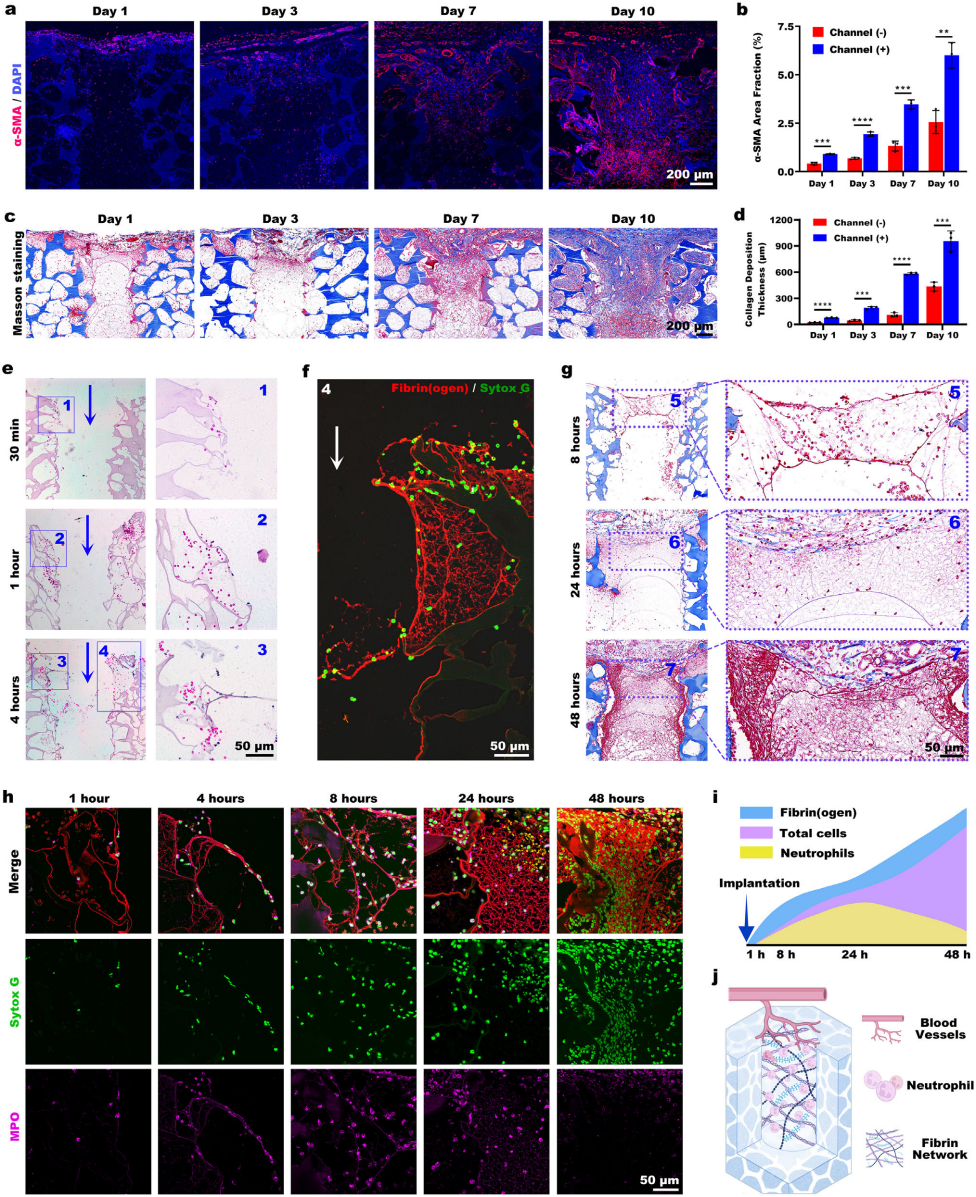
Immune complexes occupy hollow channels prior to vascular ingrowth. a) Vascular distribution within the scaffolds was revealed by time‐course analysis of α‐SMA immunofluorescence staining at 1, 3, 7, and 10 days after implantation. b) Quantification of α‐SMA⁺ area in channeled versus nonchanneled scaffolds (*n* = 3 biological replicates). c) Masson's trichrome staining of collagen deposition at corresponding time points. d) Statistical analysis of collagen deposition thickness in channeled versus nonchanneled scaffolds (*n* = 3 biological replicates). e) H&E staining showing early‐phase tissue responses at 30 min, 1 h, and 4 h. f) Immunofluorescence stain for fibrinogen with Sytox Green nuclear counterstain. g) Masson's trichrome staining at 8, 24, and 48 h postimplantation. h) Fibrinogen and myeloperoxidase (MPO) immunofluorescence costaining at 1, 4, 8, 24, and 48 h, with nuclei counterstained by Sytox Green. i) Schematic of early‐stage changes in channel content. j) Schematic of immune complexes occupying the channel structure prior to vascular ingrowth. Statistical analyses were carried out using a two‐way ANOVA and a multiple comparisons test for (b) and (d). ^**^ *p* < 0.01, ^***^ *p* < 0.001, and ^****^ *p* < 0.0001 indicate statistical significance.

To investigate how channel structures accelerate vascularization and tissue integration, we examined the angiogenesis and extracellular matrix (ECM) deposition into channel‐incorporated porous scaffolds. The scaffolds were implanted subcutaneously in rats and harvested at predetermined endpoints (1, 3, 7, and 10 days postoperation). Vascular infiltration was assessed using α‐SMA immunofluorescence staining (**Figure** **3**a). Both porous and channeled structures exhibited centripetal vascular invasion patterns, advancing from peripheral to central regions. Channeled scaffolds exhibited accelerated vascular infiltration kinetics compared to the porous controls. Quantification of α‐SMA^+^ areas (Figure 3b) revealed that channels significantly enhanced neovascularization. Similarly, Masson's trichrome staining (Figure 3c) revealed increased collagen deposition and organization in the channeled structures. Statistical analysis of collagen deposition thickness showed that channels significantly accelerated collagen deposition and overall scaffold integration (Figure 3d).

Notably, by day 1, a cell‐populated network scaffold had completely occupied the channels prior to vascular ingrowth. Over time, as vascular and tissue ingrowth extended deeper into the channels, the network scaffold degraded and receded. By day 10, the network scaffold had been completely replaced by vascularized tissue. These observations imply that the transitional network scaffold potentially facilitates directional vascular and tissue growth by providing a “track” for ingrowth.

To elucidate the formation, composition, and main functional role of this temporary network scaffold, we conducted sequential observations at various time points after implantation (30 min to 4 h). 30 min postimplantation, H&E staining revealed sparse erythrocyte infiltration along the walls of the superficial channels (Figure 3e). By 1 h, erythrocyte infiltration had progressively extended, and occasional nucleated cells were observed within the channels. After 4 h, the infiltration of both erythrocytes and nucleated cells had intensified further and penetrated deeper regions. Notably, a nascent network scaffold with associated cellular infiltrates was present in the central channel area at this stage.

Immunofluorescence staining for fibrinogen identified fibrinogen as the primary component of the channel wall and central fibrous networks (Figure 3f). Infiltrating cells were found to adhere to this fibrin scaffold. Masson's trichrome staining at 8 h revealed a loose, red‐stained network in the superficial regions of the channels (Figure 3g). By 24 h, the network expanded to fill the entire channel and became denser. Further densification occurred by 48 h, meaning that the provisional scaffold developed progressively from the superficial to the deep regions while densifying. The results of the fibrinogen and myeloperoxidase (MPO) immunofluorescence costaining are shown in Figure 3h. 1 h after the start of the experiment, fibrinogen deposition was observed on the channel walls, accompanied by minimal neutrophil infiltration. By 4 h, fibrin formed a network structure on the channel walls. Neutrophils adhered to the fibrin strands that extended toward the central channel region. 8 h after implantation, a loose fibrin network had formed in the central channel area, accompanied by increased neutrophil infiltration. After 24 h, the fibrin network in the central channel region had densified, accompanied by increased neutrophil recruitment. By 48 h, the fibrin network structure had further densified, with total cellular infiltration continuing to increase and neutrophil infiltration decreasing significantly. In summary, the biological scaffold formed rapidly in the early‐stage channels and consists primarily of a fibrin‐based network and its adherent cells. The infiltration and growth of the network scaffold involves progressive cell penetration into the channels, followed by their integration with the fibrin to form the biological network structure.

Over a period ranging from 1 to 24 h, the total number of neutrophils infiltrating the channel increased continuously. Although their proportion decreased over time, neutrophils still accounted for over 80% of the total cell count and remained the predominant cell type within the fibrin network after 24 h (Figure 3i). Figure 3j depicted this transient structure, which consists predominantly of neutrophil‐fibrin composites and represents the critical transitional structure preceding vascular ingrowth into the channels.

### 2.3 Fibrinogen Coordinates Platelet‐Mediated Neutrophil Recruitment into Channels

**Figure 4:**
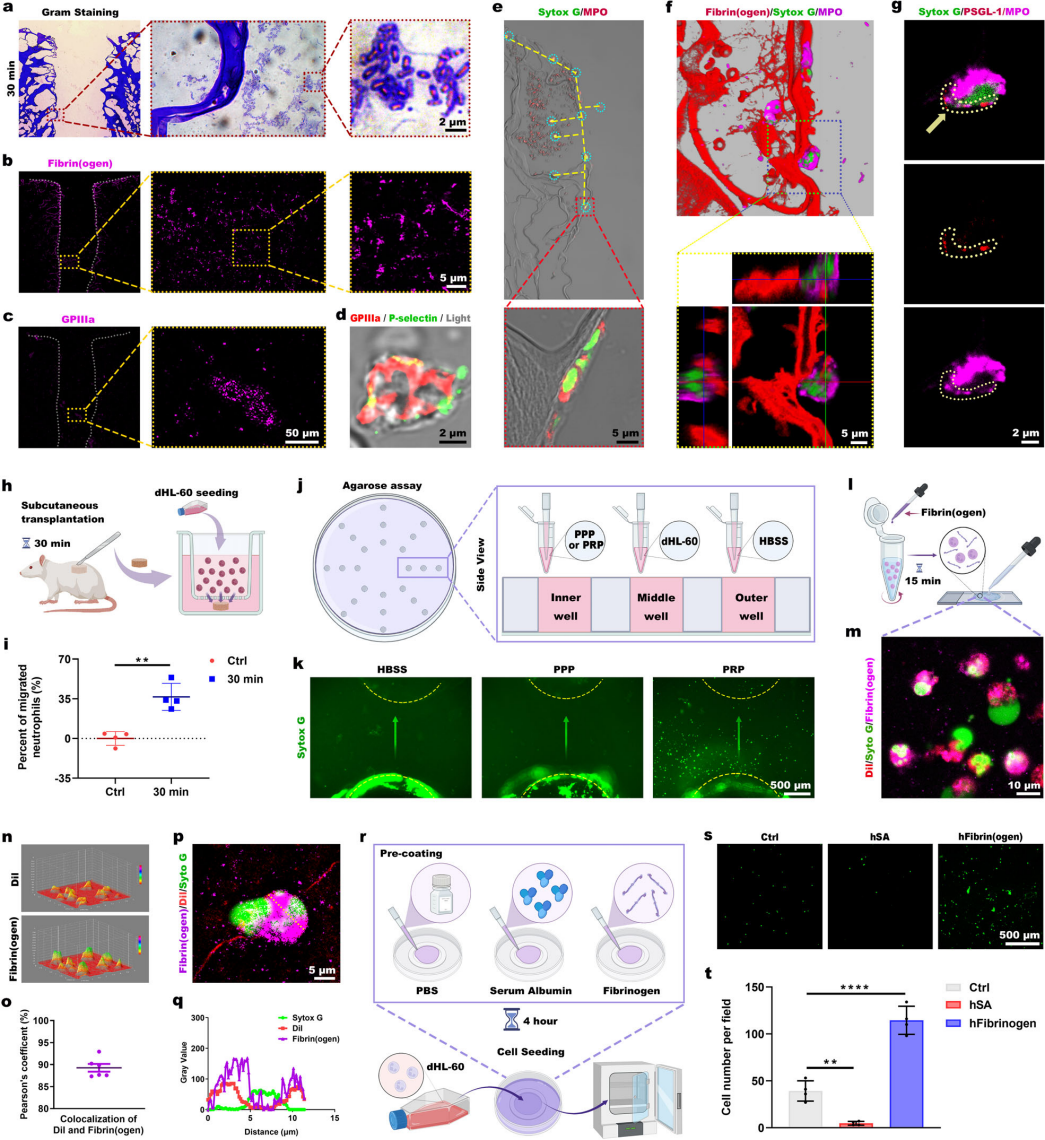
Platelets and fibrinogen collaboratively drive neutrophil recruitment into channels. a) Gram staining for histological observation of the channel structure at 30 min. b) Fibrinogen infiltration in channel structure. c) GPIIIa immunofluorescence staining to observe platelet infiltration. d) Activated platelets (GPIIIa⁺/P‐selectin⁺) in the channel. e) MPO immunofluorescence staining of the channel structure at 1 h. f) 3D reconstruction including orthogonal sections (x‐z/y‐z), showing fibrinogen‐MPO costaining, with nuclei counterstained by Sytox Green. g) PSGL‐1 and MPO immunofluorescence costaining. h) Schematic of Transwell system: The lower chamber contains in vivo‐primed scaffolds (30 min subcutaneous implantation), and the upper chamber contains dHL‐60. i) Quantified migrated neutrophils at 4 h (*n* = 4 biological replicates). j) Schematic of under‐agarose migration assay: Inner wells: PPP/PRP; outer wells: HBSS control; middle wells: dHL‐60 cells for 24 h. k) Sytox Green‐stained nuclei were imaged to observe migration. l) Schematic of fibrinogen deposition assay: A fibrinogen solution and a dHL‐60 cell suspension were mixed for 15 min before smearing. m) Fibrinogen was costained with Dil‐labeled dHL‐60 membranes and Sytox Green‐labeled nuclei. n) ImageJ‐generated fluorescence heat maps of Dil and fibrinogen. o) Pearson's colocalization coefficient (*n* = 6 biological replicates). p,q) Cross‐sectional analysis of fibrinogen and dHL‐60 colocalization. r) Schematic of neutrophil adhesion assay: Plates were precoated with PBS, hSA, or hFibrinogen, and dHL‐60 cells were seeded on them for 1 h. s) Representative images of adhered dHL‐60. t) Quantification of adhered dHL‐60 (*n* = 4 biological replicates). Significance was determined by an unpaired *t*‐test i) and a one‐way ANOVA with a Tukey's post hoc correction t). ^**^ *p* < 0.01, ^****^ *p* < 0.0001 indicate statistical significance.

Neutrophils are the predominant cell type present during the initial stage of channel structure occupation (Figure 3h). However, the spatiotemporal regulation of this process and its role in angiogenesis remain unclear. To capture the initiation phase, the channeled scaffolds were explanted 30 min after implantation, before significant cellular infiltration occurred. Gram staining revealed purple‐stained rod‐like and filamentous structures in the central channel regions (**Figure** **4**a). Fibrinogen immunofluorescence staining confirmed that these structures were fibrinogen deposits (Figure 4b). The conversion of fibrinogen to fibrin typically indicates the activation of the coagulation cascade. Consistent with this, immunofluorescence staining of platelet membrane glycoprotein IIIa (GPIIIa) (Figure 4c) and P‐selectin (Figure 4d) revealed platelets activation within the deeper regions of the channels. Within 1 h of implantation, the first wave of cells reached the scaffold surface and channel walls. MPO immunofluorescence staining identified these pioneer cells as neutrophils (Figure 4e). Costaining for fibrinogen and MPO (Figure 4f) revealed that the neutrophils were adhered to the fibrinogen matrices. Channel‐infiltrating neutrophils exhibited polarized morphology rather than symmetrical shapes. P‐selectin glycoprotein ligand‐1 (PSGL‐1) and MPO costaining (Figure 4g) revealed PSGL‐1 localization in the uropod. These findings suggest that neutrophil recruitment into channels may be driven by PSGL‐1 interactions with P‐selectin, which is expressed by activated platelets.^[^ ^16^, ^17^, ^18^, ^19^, ^20^ ^]^

We designed a Transwell assay to evaluate the chemotactic effect of the channel contents on neutrophil migration 30 min postimplantation, as shown in Figure 4h. After 4 h of coculture, 36.67% ± 10.33% of the seeded neutrophils migrated toward to the lower chamber containing the subcutaneously implanted scaffolds (Figure 4i). The channel contents were found to significantly enhance neutrophil migration, indicating their chemotactic activity.

To determine whether this chemotactic effect was mediated by fibrinogen or activated platelets, we performed an under‐agarose migration assay (Figure 4j). Neutrophils were cocultured with platelet‐poor plasma (PPP), platelet‐rich plasma (PRP), or Hanks' balanced salt solution (HBSS) for 24 h. Sytox Green nuclear staining revealed distinct migration patterns (Figure 4k). Neutrophils migrated significantly toward the PRP‐containing wells. However, they remained stationary in the HBSS control group and the PPP group. These results suggest that activated platelets primarily mediate the chemotactic activity, with negligible contribution from fibrinogen.

Although fibrinogen does not drive chemotaxis (Figure 4k), we investigated its potential role in neutrophil adhesion, considering the known interactions of αMβ2 integrins.^[^ ^21^, ^22^, ^23^ ^]^ Neutrophils were incubated with fibrinogen for 15 min prior to smear preparation (Figure 4l). The neutrophil nuclei were labeled with Syto Green, the cell membranes with Dil, and the fibrinogen was detected by immunofluorescence staining. As shown in Figure 4m, fibrinogen‐positive areas overlapped with regions of the cell membrane. ImageJ analysis revealed significant colocalization between fibrinogen immunostaining and DiI‐labeled neutrophil membranes (Figure 4n), with a Pearson's coefficient of ≈85% (Figure 4o). Typical neutrophil cross‐sectional analysis further confirmed substantial overlap between fibrinogen and neutrophil membrane fluorescence signals (Figure 4p,q). These findings confirm fibrinogen deposition on neutrophil surfaces.

We examined the role of fibrinogen in neutrophil adhesion by coating culture dishes with: i) human serum albumin (hSA), ii) phosphate‐buffered saline (PBS), or iii) human fibrinogen (hFibrinogen) (Figure 4r). After allowing neutrophils to adhere to the differently coated surfaces for 1 h, Sytox Green nuclear staining and fluorescence microscopy were performed (Figure 4s). We found that hSA inhibited neutrophil adhesion while fibrinogen enhanced it. Statistically significant differences were observed among the three groups (Figure 4t). In summary, hollow channels facilitate the rapid perfusion of fibrinogen and platelets, particularly in deep core regions. Activated platelets mediate neutrophil recruitment, and fibrinogen deposition promotes neutrophil adhesion. These processes work together to drive neutrophil migration into the channels, resulting in infiltration of the deep region.

### 2.4 Fibrin ogen Stimulates Neutrophil Activation and NETs Formation

**Figure 5:**
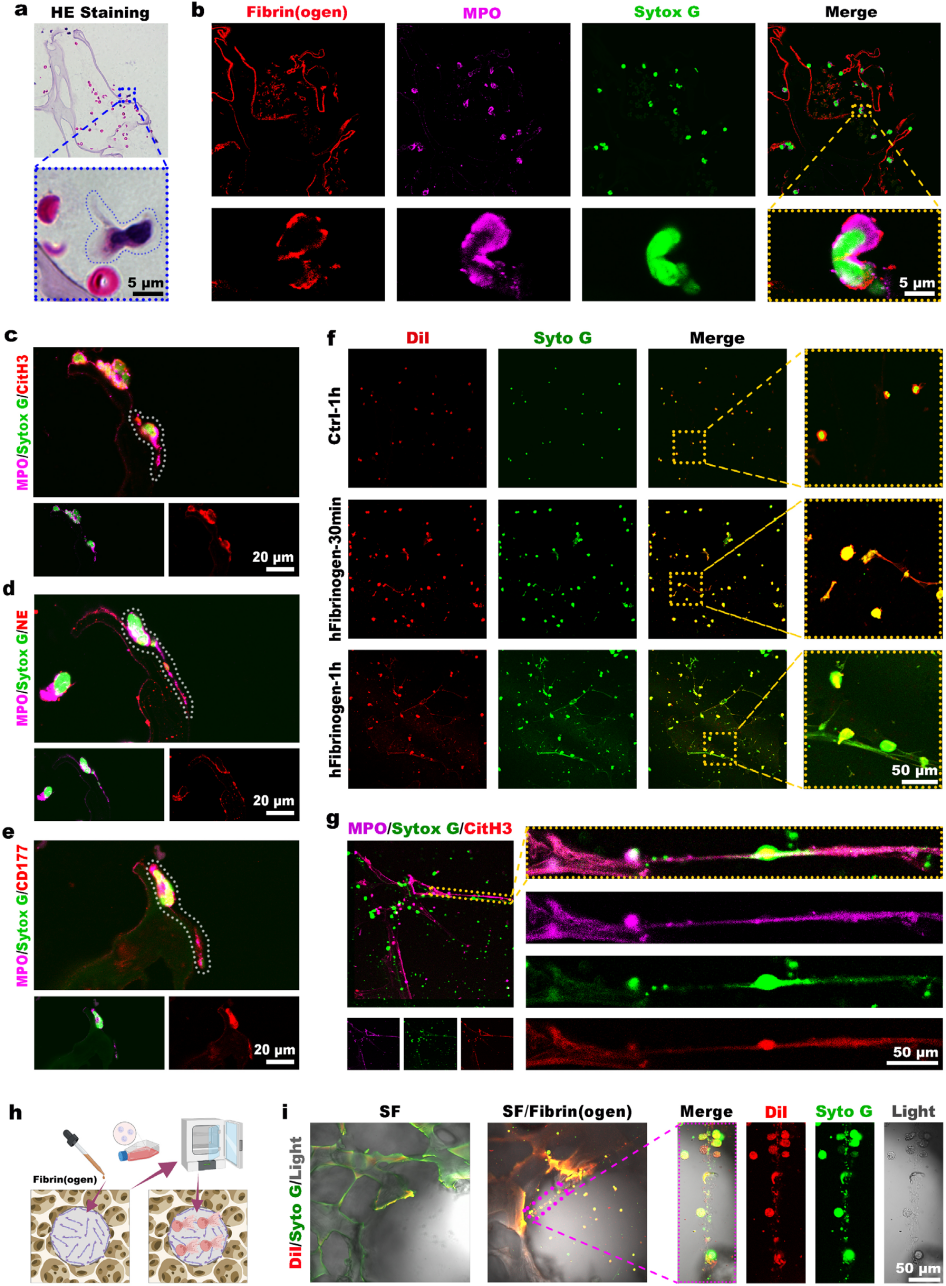
Neutrophil activation and NETs formation mediated by fibrinogen. a) H&E staining of rat subcutaneous scaffolds 1 h postimplantation. b) Immunofluorescence costaining of fibrinogen and MPO. c–e) Serial paraffin sections were subjected to immunofluorescence costaining for: c) MPO and CitH3, d) MPO and NE, e) MPO and CD177. Nuclei were counterstained with Sytox Green. f) dHL‐60 cells were seeded onto PBS/hFibrinogen‐coated dishes. Morphological changes were observed by labeling cell membranes with Dil and nuclei with Sytox Green. g) Immunofluorescence costaining for MPO and CitH3 in dHL‐60 after 1 h, with nuclei counterstained with Sytox Green. h) 3D coculture scheme: Fibrinogen‐preadsorbed scaffolds were seeded with dHL‐60 cells for 1 h. i) Cell membranes were labeled with Dil, and the nuclei with Syto Green.

At 1 h after scaffold implantation, H&E staining (**Figure** **5**a) revealed nuclear externalization in cells infiltrating the channels. Immunofluorescence costaining of MPO and fibrinogen (Figure 5b), combined with Sytox Green nuclear staining, revealed fibrinogen deposition on neutrophil surfaces, and chromatin externalization. This indicates the formation of NETs. Costaining of MPO with citrullinated histone H3 (CitH3), neutrophil elastase (NE), and CD177 (Figure 5c–e) showed that NETs formation was caused by CD177^+^ neutrophils with activated effector functions.

In addition to its adhesive and structural functions, fibrinogen acts as a potent immunomodulator that directly activates neutrophil effector functions.^[^ ^24^ ^]^ To investigate its role in neutrophil activation and NETs release, we performed in vitro experiments in which we seeded neutrophils onto fibrinogen‐coated culture dishes (Figure 5f). Untreated neutrophils maintained a spherical morphology with lobulated nuclei. Within 30 min of fibrinogen exposure, the neutrophils significantly enlarged and exhibited DNA externalization. After 1 h, significant cell elongation indicated NETs formation, which was confirmed by CitH3 and MPO immunofluorescence staining (Figure 5g).

Next, we developed a 3D coculture system to model the effect of fibrinogen on the neutrophil into channels (Figure 5h). After 1 h of culturing, the untreated scaffolds showed minimal neutrophil observed. In contrast, hFibrinogen‐pretreated scaffolds demonstrated significantly increased neutrophil attachment and channel occupation (Figure 5i). The presented results revealed a decisive all‐or‐none outcome: control channels remained empty, while fibrinogen‐coated channels were filled with extensive NETs networks, as confirmed by Syto Green‐labeled DNA externalization. This phenomenon confirmed that the deposition of fibrinogen on the scaffold surface is crucial for activating neutrophils to form NETs that infiltrating the hollow channel.

### 2.5 Directional Ingrowth of NETs‐Fibrin Biological Scaffold into Channels

**Figure 6:**
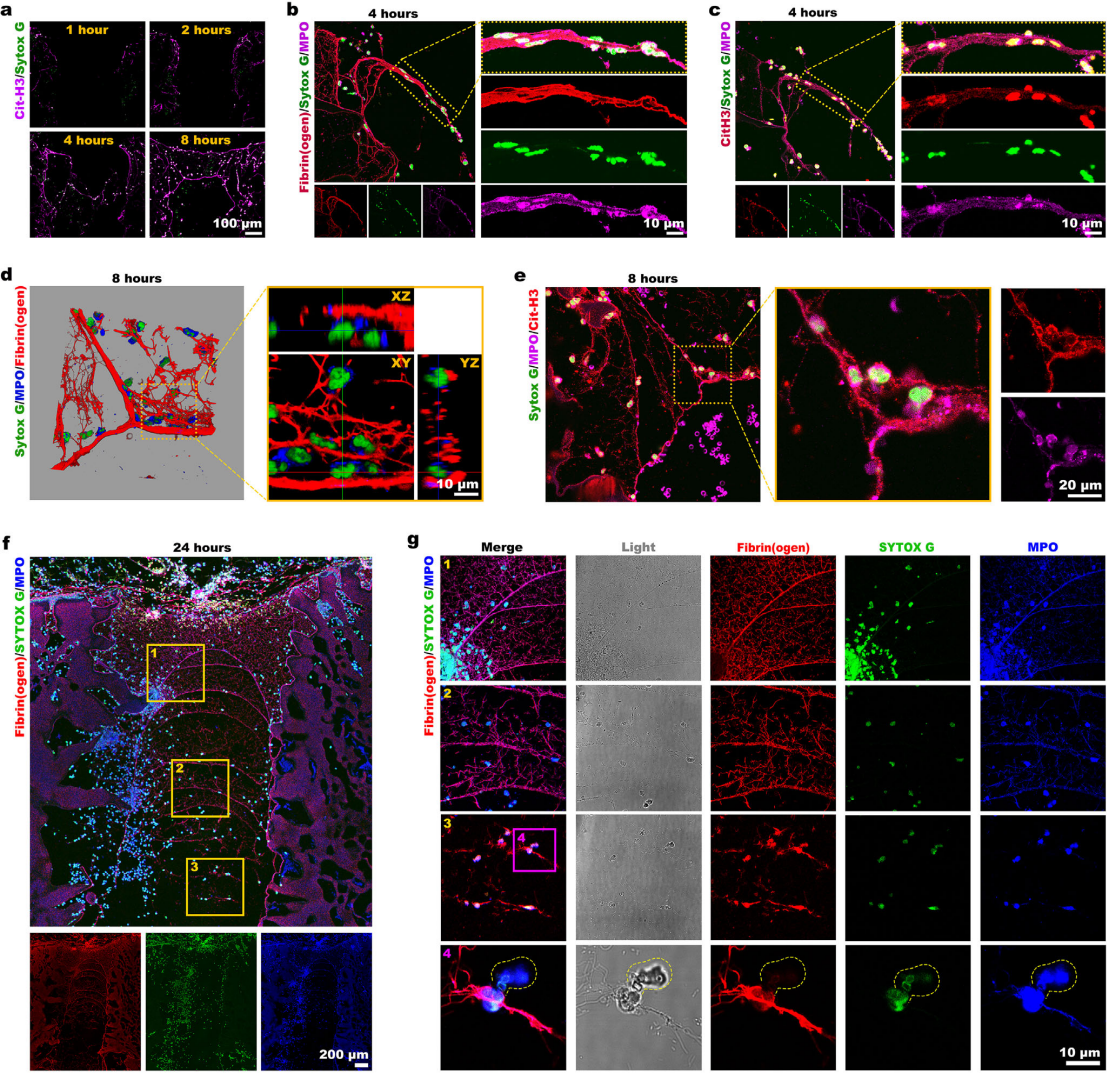
Infiltration and occupation of the channel structure by NETs‐fibrin biological scaffolds. a) CitH3 immunofluorescence staining at 1, 2, 4, and 8 h postimplantation, with the nuclei counterstained using Sytox Green. b,c) Immunofluorescence costaining was conducted for b) fibrinogen and MPO, and c) CitH3 and MPO at 4 h, with the nuclei counterstained using Sytox Green. d) 3D reconstruction including orthogonal sections (x‐z/y‐z), showing fibrinogen‐MPO costaining at 8 h postimplantation. e) Immunofluorescence costaining for CitH3 and MPO at 8 h postimplantation, with the nuclei counterstained using Sytox Green. f,g) Immunofluorescence costaining of fibrinogen and MPO at 24 h, with the nuclei counterstained using Sytox Green.

In the early stages, the provisional network scaffold in channels consists primarily of fibrinogen, and infiltrating neutrophils are the dominant cell population. Since the interaction between neutrophils and fibrinogen triggers the release of NETs, we hypothesized that NETs are involved in forming the provisional network scaffold. Immunofluorescence staining revealed that CitH3^+^ NETs advanced progressively from channel periphery to the central region over the course of 1–8 h, forming an interconnected network (**Figure** **6**a).

Notably, the spatiotemporal progression of NETs precisely paralleled the maturation of the fibrin network. Multiplex coimmunofluorescence staining at 4 h revealed that the developing scaffold strands contained fibrinogen with adherent neutrophils (Figure 6b for MPO/fibrinogen, Figure 6c for MPO/CitH3, and Figure S3 (Supporting Information) for MPO/CD177). These CD177^+^ neutrophils exhibited CitH3 positivity, confirming the formation of NETs. 3D reconstruction of confocal images taken from MPO/fibrinogen immunofluorescence costaining (Figure S4, Supporting Information) demonstrated that DNA‐releasing neutrophils were interwoven with fibrin into strand‐like structures. By 8 h, 3D‐reconstructed confocal images taken from MPO/fibrinogen immunofluorescence costaining (Figure 6d), showed that the channel network scaffold consisted of fibrin matrices and neutrophils. MPO/CitH3 immunofluorescence costaining (Figure 6e) revealed the incorporation of neutrophil‐derived NETs into the forming scaffolds. At 24 h, MPO/fibrinogen immunofluorescence costaining revealed channels filled with fibrin networks incorporating neutrophils, with characteristic fibrinogen deposition on neutrophil surfaces and concurrent MPO, and chromatin externalization (Figure 6f,g). These results definitively establish NETs as core scaffold components alongside fibrin. The NETs‐fibrin scaffold exhibited distinct spatial organization, with maximum density in superficial regions and decreasing density toward deeper areas, where the network appeared most porous.

### 2.6 NETs Recruit Macrophages and Provide Guidance Pathways within Channels

**Figure 7:**
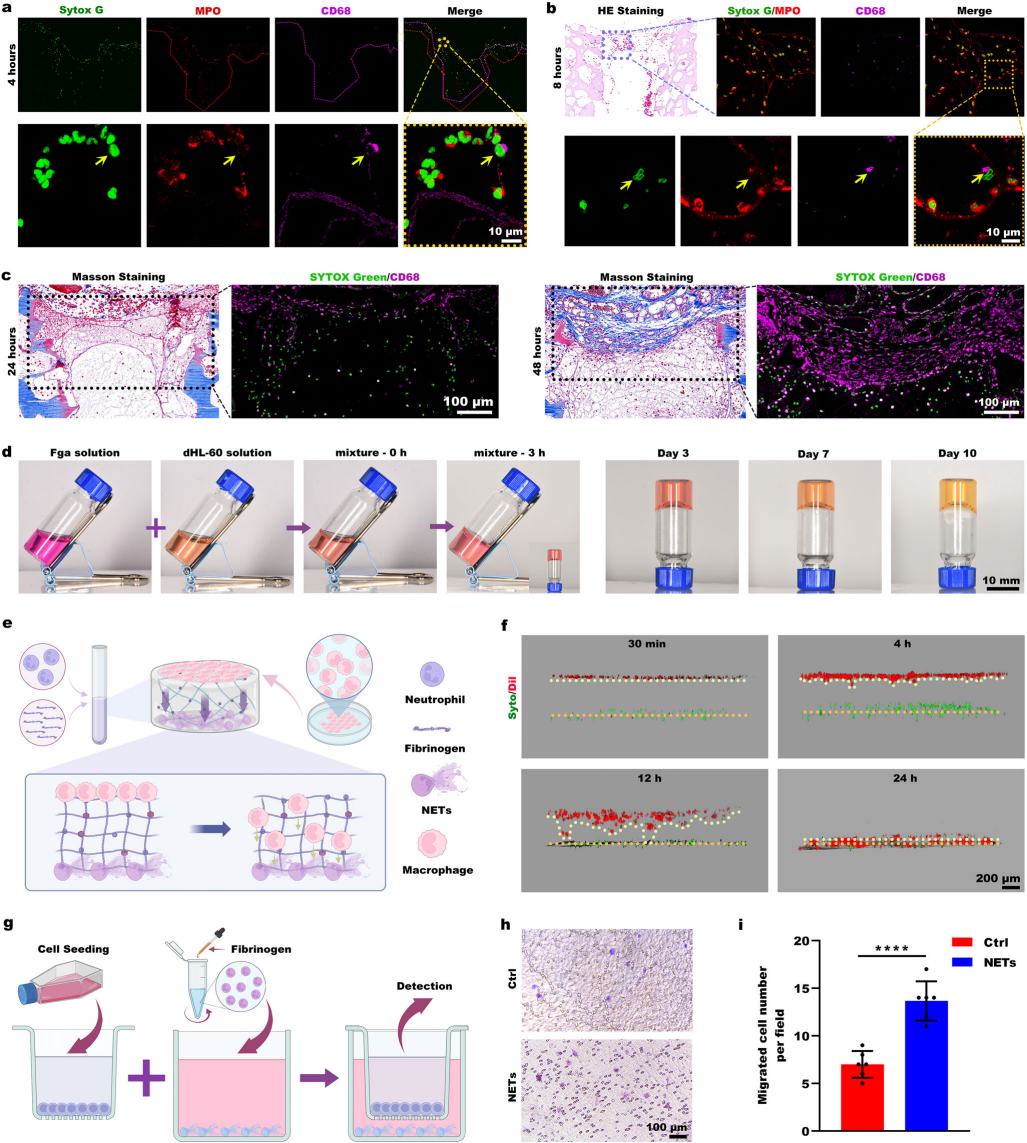
NETs drive macrophage infiltration into channel structures. a) MPO⁺ neutrophils and CD68⁺ macrophages in subcutaneously implanted scaffolds at 4 h, with nuclei counterstained using Sytox Green. b) H&E staining at 8 h postimplantation, along with immunofluorescence costaining of MPO and CD68, with Sytox Green nuclei counterstain. c) Left: Masson's trichrome staining at 24, 48 h; Right: CD68⁺ macrophages in serial sections. d) Gelation assay: A mixture of fibrinogen solution and dHL‐60 cell suspension transitioned from a liquid to a gel state within 3 h. e) Schematic showing the design of the 3D coculture experiment to assess the chemotactic effect of NETs on THP‐1 cells. f) THP‐1 cells (Dil, red) were seeded on the surface of NETs (Syto Green, green)/fibrin gel, and imaged at specified time points. g) Schematic of the Transwell assay for evaluating the chemotactic effect of NETs on THP‐1 cells. h) Migrated THP‐1 cells (crystal violet⁺) at 24 h. i) Quantification and statistical analysis of migrated THP‐1 cells (*n* = 6 biological replicates). Data were analyzed using unpaired *t*‐tests, with statistical significance defined as ^****^ *p* < 0.0001.

The infiltration of NETs‐fibrin biological scaffolds into channels is accompanied by substantial cellular infiltration. In addition to neutrophils, macrophages are another critical cell population. 4 h postimplantation, immunofluorescence staining revealed sparse CD68^+^ macrophages within the channels (**Figure** **7**a). These macrophages exhibited significantly more limited distribution than MPO^+^ neutrophils. By 8 h, CD68 immunofluorescence staining revealed that macrophages had penetrated the core regions of the loose network scaffold perfusing the channels (Figure 7b). Figure 7c shows that a prolonged implantation time of up to 24 and 48 h, Masson's trichrome staining combined with CD68 immunofluorescence staining revealed deeper macrophage penetration and an increased number of macrophages within the channel's network scaffold. These results demonstrate a distinct migration pattern toward deeper regions. These findings suggest that the NETs‐fibrin biological scaffold provides structural support and chemotactic recruitment for the infiltration and migration of macrophages within the channels.

In vitro studies showed that mixing fibrinogen solution with dimethyl sulfoxide (DMSO)‐induced HL‐60 driven neutrophil like (dHL‐60) cells at 37 °C for 3 h induced gelation and formed stable NETs‐fibrin hydrogels that maintained structural integrity for 10 days (Figure 7d). To evaluate macrophage recruitment, NETs‐fibrin hydrogels were prepared, and then macrophages were seeded on their surface (Figure 7e). Staining the nuclei of the dimet (dHL‐60) cells with Syto Green and the membranes of the THP‐1 cells with Dil revealed that, at 30 min postseeding, the macrophages remained ≈400 µm from the NETs layer. By 4 h, initial migration toward the NETs layer was observed. At 12 h, substantial migration occurred, reducing the interlayer distance. Complete migration to the NETs layer was achieved within 24 h (Figure 7f). Transwell assays (Figure 7g) confirmed NETs‐mediated chemotaxis. Quantitative analysis revealed significantly increased macrophage migration through the pores compared to the controls (Figure 7h,i). These findings establish that the early host response within the channel involves the directional infiltration of a NETs‐fibrin scaffold laden with immune cells. Neutrophils mediate rapid but transient channel perfusion in this process, and NETs subsequently recruit macrophages that exhibit delayed but sustained infiltration.

### 2.7 Macrophage‐Derived VEGF Drives Neovascular Ingrowth into Hollow Channels

**Figure 8:**
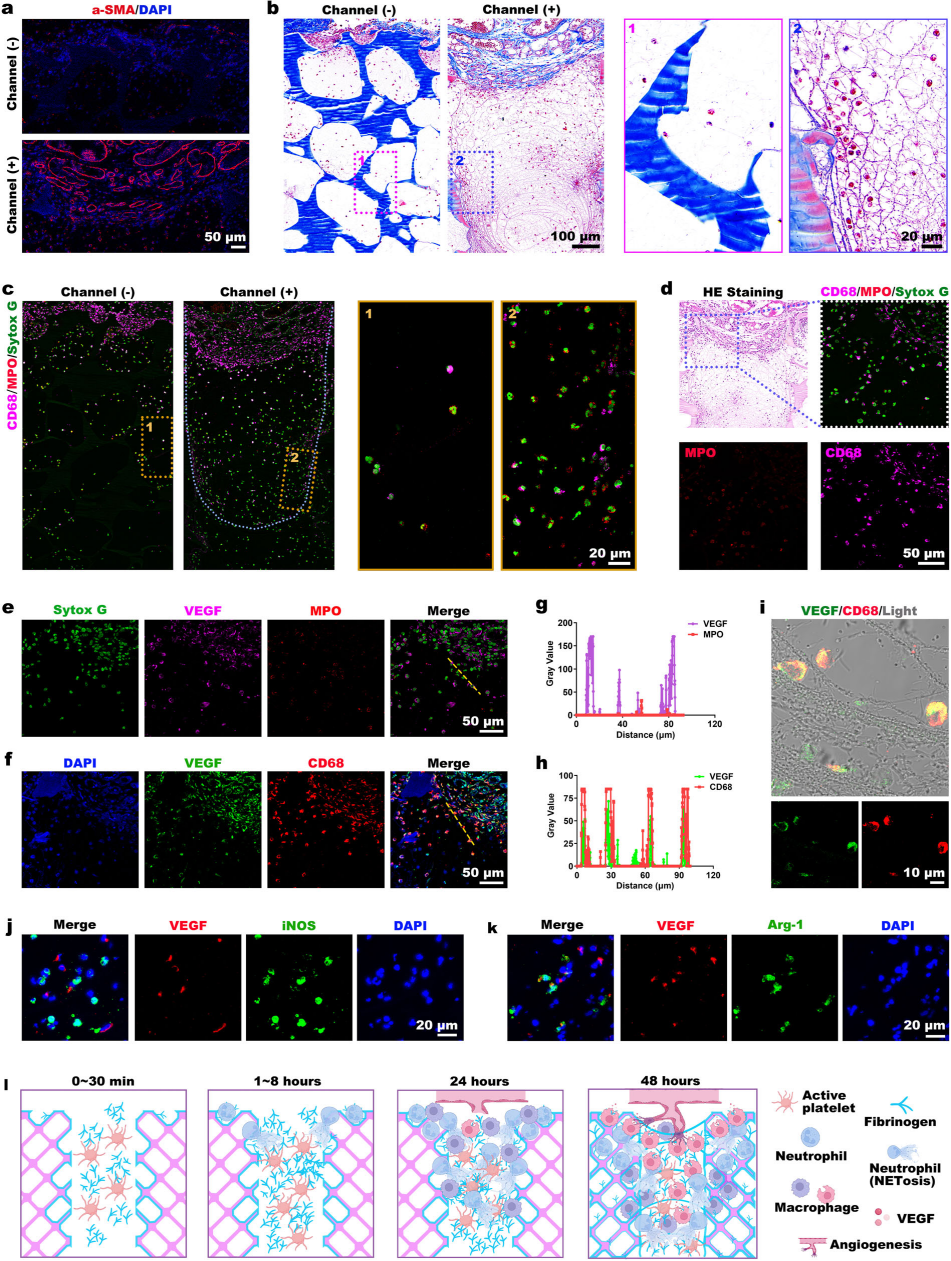
Macrophage drives angiogenesis via VEGF secretion. a) α‐SMA⁺ vessels in subcutaneously implanted scaffolds at 48 h (DAPI‐counterstained nuclei). b) Masson's trichrome staining for histological observation. c) Immunofluorescence costaining of MPO⁺ neutrophils and CD68⁺ macrophages in channeled/nonchanneled structures, with nuclei counterstained using Sytox Green. d) Histological observation of the superficial channel region via H&E staining, followed by immunofluorescence costaining of MPO and CD68, with nuclei counterstained using Sytox Green. e) Immunofluorescence costaining of VEGF and MPO, with nuclei counterstained using Sytox Green. f) Immunofluorescence costaining of VEGF and CD68, with DAPI‐counterstained nuclei. g,h) Representative cross‐sectional colocalization analysis of g) VEGF/MPO and h) VEGF/CD68 immunofluorescence staining. i) Immunofluorescence costaining of VEGF and CD68 in the deep channel region. j) Immunofluorescence co‐staining of VEGF and iNOS. k) Immunofluorescence costaining of VEGF and Arg‐1. l) Schematic diagram illustrating the biological mechanism of rapid neoangiogenesis initiation in channel structure.

Immunofluorescence staining of α‐SMA (**Figure** **8**a) showed that the channeled constructs had more new blood vessels than the porous controls at 48 h postimplantation. Masson's trichrome staining (Figure 8b) showed the formation of a more mature network scaffold within the channels. Immunofluorescence costaining of MPO and CD68 (Figure 8c) revealed significantly greater cellular infiltration in the channeled structures than in the porous controls. This infiltration comprised enhanced neutrophil and macrophage populations. Complementary H&E staining revealed that macrophages predominanted at the channel‐guided neovascularization front, with only residual neutrophils present (Figure 8d).

Studies of VEGF‐A colocalization revealed a specific correlation with CD68^+^ macrophages rather than MPO^+^ neutrophils (Figure 8e–h). High VEGF‐A expression was exclusively identified in CD68^+^ macrophages in deep channel regions (Figure 8i). Since VEGF‐A is a pivotal regulator of angiogenesis,^[^ ^25^ ^]^ these findings establish macrophages as the primary source of this key angiogenic factor that drives polarized vascular ingrowth. To characterize the macrophage phenotype, we performed immunofluorescence costaining of VEGF‐A with both inducible nitric oxide synthase (iNOS) and arginase 1 (Arg‐1). Preliminary results (Figure 8j,k) showed that most VEGF‐A‐positive macrophages coexpressed the M2 marker Arg‐1, while few expressed the M1 marker iNOS. Consistent with this observation, our findings collectively suggest that the VEGF‐A‐secreting macrophages which drive angiogenesis are predominantly of the M2 phenotype.

To determine whether NETs are essential for channel‐mediated vascularization, we conducted neutrophil depletion experiments. Following 14 days of subcutaneous implantation in rats, H&E staining (Figure S5a, Supporting Information) revealed robust neovascularization and tissue ingrowth within the channels in both control and neutrophil‐depleted groups. The efficacy of depletion was confirmed by Ly6G immunofluorescence staining and quantitative analysis (Figure S5b,c, Supporting Information), which showed a significant reduction in neutrophil infiltration, validating the success of our intraperitoneal anti‐Ly6G antibody protocol. While CD31 immunofluorescence staining (Figure S5d, Supporting Information) confirmed the presence of new blood vessels, quantitative analysis of the CD31^+^ area (Figure S5e, Supporting Information) showed only a slight and statistically insignificant reduction in the depletion group. The results were illuminating: while depletion was effective, it led to only a modest, statistically insignificant reduction in overall vascularization. Therefore, our findings indicate that while neutrophil depletion does not abrogate channel‐mediated vascularization, they collectively lead us to conclude that the integrated NETs‐fibrin‐macrophage complex, rather than NETs alone, that plays a critical role in rapidly guiding vascular ingrowth into the channels.

In summary, the biological mechanism by which hollow channel structures guide rapid and directional vascular and tissue ingrowth is as follows (Figure 8l): Immediately after implantation, the scaffold's channel structure guides the rapid infiltration of fibrinogen and platelets throughout the channels, including the deep core regions. Subsequently, neutrophils derived from local tissues or extravasated from nearby blood vessels migrate into the channels, likely via scanning activated platelets through the P‐selectin/PSGL‐1 axis. Upon engagement with fibrinogen, their effectors activate and release NETs. The released NETs then recruit macrophages to migrate into the channels. The interweaving of the NETs with fibrinogen forms a 3D network scaffold that rapidly fills the entire hollow channel structure. Together with resident macrophages, they form an immune‐complex provisional matrix within the channels, wherein M2 macrophages act as the primary source of VEGF‐A and critically contribute to initiating neovascularization. This process subsequently coordinates fibroblast migration and new collagen matrix deposition. Ultimately, accelerated vascularization and tissue remodeling of the scaffold are achieved.

### 2.8 Blood Clot Preloading Potentiates Scaffold Vascularization

**Figure 9:**
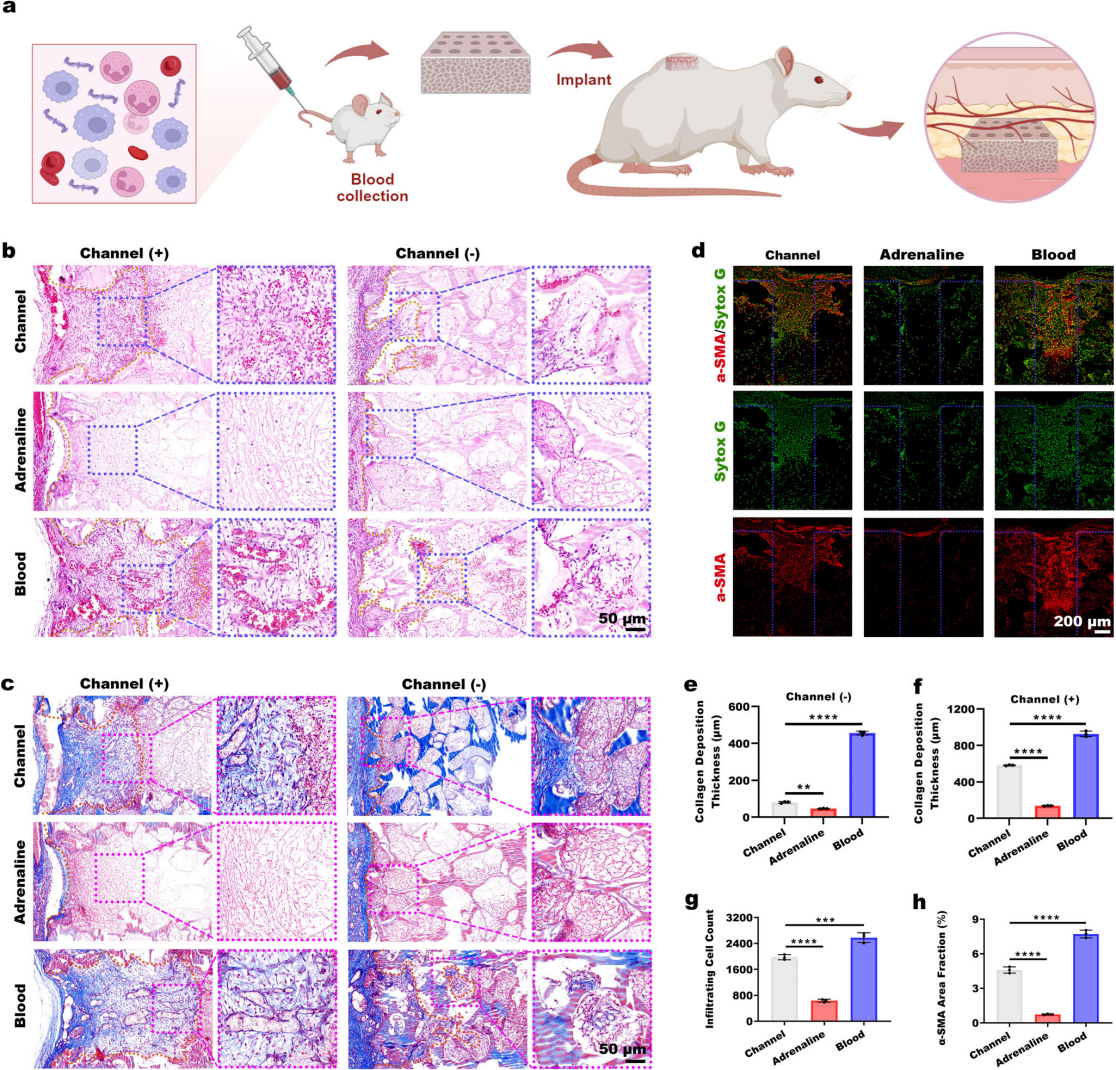
Preloaded blood clot synergizes with channel structures to enhance vascularization and bone regeneration. a) Schematic of experimental designs evaluating vascularization in rat subcutaneous implantation model. b) H&E staining and c) Masson's trichrome staining at 7 days after implantation, showing tissue ingrowth into channeled/nonchanneled structures. d) α‐SMA⁺ vessels in scaffolds with Sytox Green‐counterstained nuclei. e,f) Statistical analysis of collagen deposition thickness within channeled/nonchanneled regions at 7 days postimplantation (*n* = 3 biological replicates). g) Quantitative analysis of total cellular infiltration and h) α‐SMA‐positive area in channeled scaffolds (*n* = 3 biological replicates). Statistical analyses were carried out using one‐way ANOVA with Tukey's post hoc correction for e–h). Significance levels: ^**^ *p* < 0.01, ^***^ *p* < 0.001, and ^****^ *p* < 0.0001.

Based on the channel‐mediated vascularization mechanism, we developed a blood clot preloading strategy for channel‐arrayed scaffolds, with the aim of enhancing the efficiency of vascularization and tissue integration in tissue‐engineered constructs. This strategy uses blood clot as a host‐derived biological scaffold whose composition resembles the channel‐induced provisional matrix.^[^ ^26^ ^]^ Vascularization efficacy was evaluated through rat subcutaneous implantation (**Figure** **9**a).

In the subcutaneous implantation studies, the control groups were the Channel group (channeled scaffolds without additional treatment) and the Adrenaline group (adrenaline‐preinfiltrated channeled scaffolds). Histological observation at 2 day postimplantation (H&E staining, Figure S6a, Supporting Information) revealed that the Adrenaline group exhibited markedly reduced cell infiltration within the channels compared to the Channel group, whereas the Blood group (scaffolds preloaded with blood clots) showed a converse effect with enhanced infiltration. This trend was confirmed at the immune cell level by MPO/CD68 immunofluorescence costaining (Figure S6b, Supporting Information), which demonstrated a concurrent decrease in both neutrophils and macrophages in the Adrenaline group and an increase in the Blood group relative to Channel group. Collectively, these findings indicate that adrenaline‐induced vasoconstriction suppresses early cellular infiltration, particularly of neutrophils and macrophages, whereas preloaded blood clot actively promote their recruitment.

Histological observations at 7 days postimplantation (H&E and Masson's trichrome staining, Figure 9b,c) revealed minimal cellular infiltration and sparse collagen deposition in the Adrenaline group, while the Channel group showed substantial tissue ingrowth, particularly in the channels. The Blood group demonstrated superior infiltration depth and cellularity in both the channel and the porous regions. α‐SMA immunofluorescence staining (Figure 9d) confirmed enhanced vascularization in the Blood group compared to the controls. A statistical analysis was conducted on the thickness of collagen deposition within the scaffolds (Figure 9e,f). The collagen deposition thickness in the porous structure region was 80.58 ± 4.59 µm in the Channel group, and it was 582.06 ± 4.71 µm in the channel structure region. In the Adrenaline group, the collagen deposition was 46.24 ± 1.56 µm thick in the porous structure region and 137.49 ± 3.19 µm thick in the channel structure region. In the Blood group, collagen deposition was 455.78 ± 10.50 µm thick in the porous structure region and 926.51 ± 30.58 µm thick in the channel structure region. Quantitative analysis of collagen deposition thickness in the channeled and porous regions confirmed the Blood group's significant advantage in tissue remodeling. Similarly, the quantitative analysis of infiltrating cell number and α‐SMA⁺ vasculature area (Figure 9g,h) revealed the Blood group's promotional effect on cellular and vascular infiltration.

### 2.9 Blood Clot Preloading Promotes Scaffold Bone Regeneration

**Figure 10:**
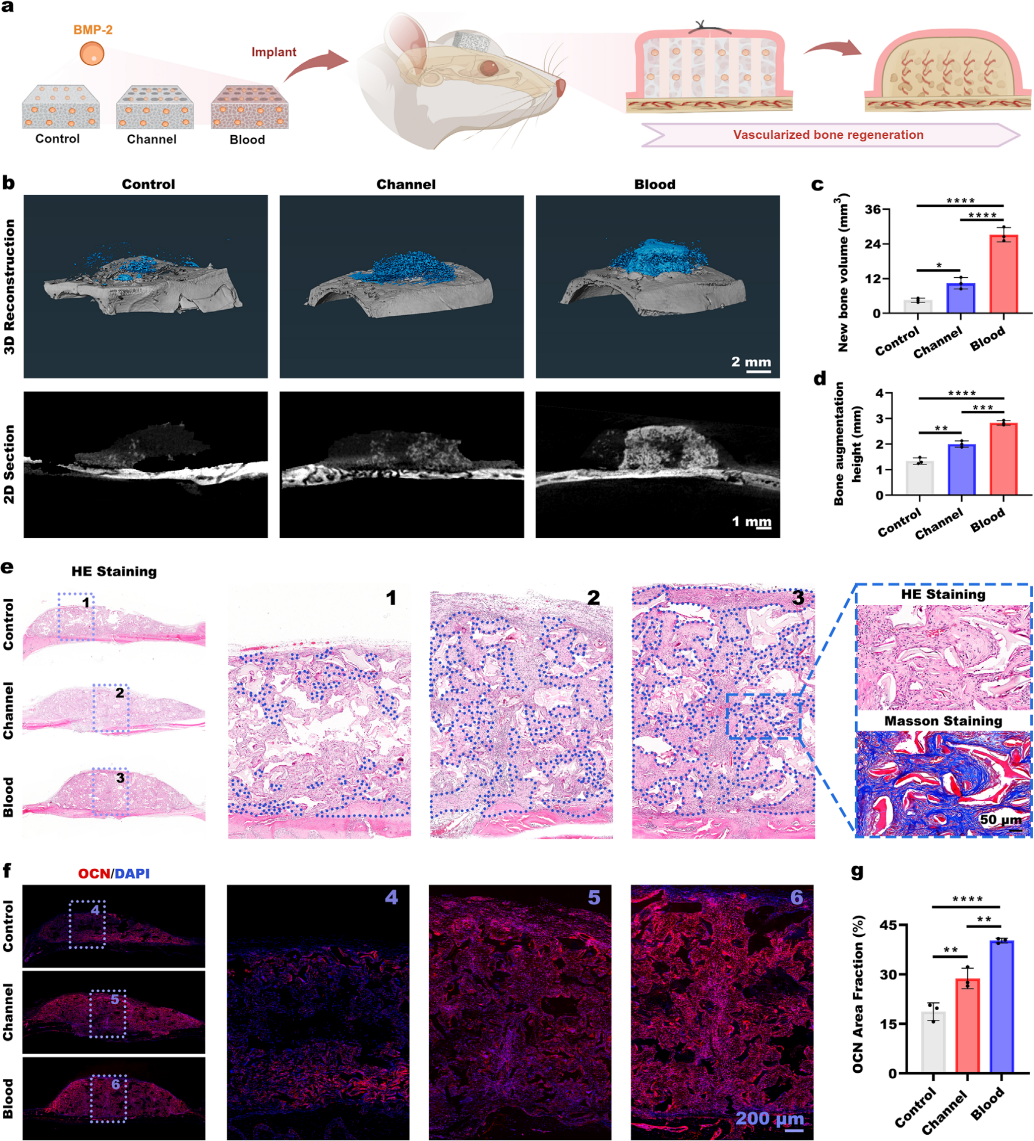
Preloaded blood clot synergizes with channel structures to enhance bone regeneration. a) Schematic of experimental designs evaluating bone regeneration in rat calvarial augmentation model. b) Micro‐CT 3D reconstruction and representative cross‐sections of newly‐formed bone 4 weeks after implanting BMP‐2‐preloaded scaffolds on rat calvarial bone. c) Statistical comparison of new bone volume and d) augmented bone height among three scaffold groups (*n* = 3 biological replicates). e) H&E staining and Masson's trichrome staining at 4 weeks after implantation. f) OCN immunofluorescence staining with DAPI‐counterstained nuclei. g) Quantification of OCN⁺ area in the three scaffold groups (*n* = 3 biological replicates). Statistical analyses were carried out using one‐way ANOVA with Tukey's post hoc correction for (c), (d), and (g). Significance levels: ^*^ *p* < 0.05, ^**^ *p* < 0.01, ^***^ *p* < 0.001, and ^****^ *p* < 0.0001.

To evaluate the osteogenic capacity of the collaborative strategy involving BMP‐2, blood clot and channel structures, rat calvarial bone augmentation model was used (**Figure** **10**a). BMP‐2/blood clot‐preloaded, channeled scaffolds (Blood group) were implanted on rat calvarial bone, alongside two control groups: BMP‐2‐preloaded nonchanneled, porous scaffolds (Control group), and, BMP‐2‐preloaded, channeled scaffolds (Channel group).

3D reconstruction images and representative sections from micro‐CT detection (Figure 10b) together with the statistical analyses (Figure 10c,d), showed that the channeled architecture significantly increased bone mineral volume and height, with the Blood group showing the most substantial improvement. These structural findings were corroborated at the tissue level. Histological observations at 4 weeks postimplantation (H&E staining, Figure 10e) revealed that channeled scaffolds better maintained tissue‐integration height than nonchanneled scaffolds, with the Blood group exhibiting the most extensive new tissue infiltration. Furthermore, Masson's trichrome staining indicated substantial collagen deposition in the Blood group.

To further evaluate osteogenic outcomes, we assessed osteoblast presence via osteocalcin (OCN) immunofluorescence staining (Figure 10f,g), which showed a clear hierarchy: channeled scaffolds supported more OCN‐positive osteoblasts than nonchanneled controls, and the Blood Group exhibited the most extensive staining. Quantitative analysis of the OCN‐positive area (Figure 10g) validated this progression, confirming that OCN expression was significantly highest in the Blood Group. These consistent findings establish that the channeled scaffold strategy, particularly when augmented with a BMP‐2‐laden blood clot, is a highly promising approach for facilitating vascularized bone regeneration.

## 3 Discussion and Conclusion

The channel structure serves as a critical design element for achieving rapid vascularization. Owing to its remarkable versatility, this channel‐based strategy has been successfully applied across diverse biomaterial systems. In synthetic polymers (e.g., poly(epsilon‐caprolactone), poly‐l‐lactic acid),^[^ ^27^, ^28^ ^]^ the incorporation of channels significantly enhances their capacity to induce early vascularization in vivo. For bio‐ceramics (e.g., calcium phosphate ceramics),^[^ ^29^, ^30^ ^]^ channels not only guide vascular ingrowth but also act synergistically with the release of bioactive ions to promote both angiogenesis and osteogenesis. Even in mechanically soft hydrogels (e.g., gelatin, collagen, fibrin), the fabrication of microchannels via techniques like microfluidics has proven effective in supporting the formation of endothelial cell‐based tubular structures.^[^ ^31^ ^]^

SF scaffolds are ideal platforms for tissue engineering due to their tunable mechanical properties, degradation rates, and superior cytocompatibility. These properties support the regeneration of diverse tissues, including vascular, dermal, osseous, cartilaginous, and neural types.^[^ ^32^, ^33^, ^34^, ^35^, ^36^ ^]^ Furthermore, the porosity and pore architecture of these scaffolds can be precisely controlled through regulated assembly methods, allowing pore sizes to be tuned from the nanoscale to several hundred micrometers. Leveraging these advantages, we fabricated porous SF scaffolds with uniform pore architectures using freeze‐drying and particle‐leaching techniques. The design featured hollow channels interconnected with the scaffold's porous network to promote cell migration and nutrient diffusion throughout the 3D construct. A minimum 1 mm‐wide porous region was preserved around each 500 µm‐diameter channel to ensure structural integrity under physiological stress and prevent adjacent channel interference. This channel‐mediated process of directional vascular ingrowth was then systematically examined.

The scaffold implantation procedure, being a surgical intervention, inevitably causes vascular damage, or increased vascular permeability. Fibrinogen, derived from vascular leakage or interstitial fluid, quickly fills the channels, reaching deep regions, and forming temporary surface matrices. In response to this, coagulation cascades are initiated, leading to a series of subsequent biological reactions. These reactions include the formation of a thrombus and the activation of immune cells. Activated platelets release bioactive factors, such as P‐selectin, which likely attract neutrophils migration into channels by interacting with PSGL‐1.^[^ ^16^, ^37^ ^]^ Fibrinogen and crosslinked fibrin networks adhere to the channel walls or luminal spaces, providing physical support for recruited neutrophils to migrate and crawl. Upon contact with fibrinogen, neutrophils become activated and release extracellular DNA to form NETs, regardless of whether they originate from adjacent tissues or extravasate from nearby vasculature. CD177, a glucose‐6‐phosphate isomerase (GPI)‐anchored surface protein that is selectively expressed on neutrophils,^[^ ^38^, ^39^ ^]^ plays a key role in regulating their migration and activation during this process.

These NETs then interweave with fibrin to form a network scaffold structure,^[^ ^40^ ^]^ the development of which proceeds bidirectionally. Initial vertical infiltration along the channel walls, from superficial to deep regions, is followed by horizontal extension toward the central lumen. Slightly later, the core region is infiltrated by a sparse but rapidly expanding network that progressively fills the entire channel space. Following their formation, these NETs scaffolds—constituting the backbone of the interwoven network—orchestrate a second wave of monocyte emigration. This long‐established causal relationship probably be mediated by the release of specific factors, such as LL‐37, azurocidin, cathepsin G (CTSG), human neutrophil peptides 1–3 (HNP1–3), and proteinase 3 (PR3).^[^ ^41^, ^42^ ^]^ Since NETs are not the only indispensable gateway for angiogenesis, we propose that the NETs‐fibrin scaffold acts as an extremely efficient accelerator of the process. It rapidly and efficiently recruits macrophages, leading to the swift formation of a proangiogenic niche within the channels. The infiltration of M2‐polarized macrophages, which secrete VEGF‐A, critically guides the initial vascular and tissue ingrowth into the channels, thereby establishing provisional structures that are subsequently replaced by rapidly growing new blood vessels and tissue—a proregenerative process supported by these macrophages.

A comparison of immune‐complex biological scaffolds in porous versus channeled regions revealed significant differences. Porous structures exhibited limited cellular infiltration and underdeveloped network scaffold formation. In contrast channeled regions demonstrated enhanced cellularity, particularly of macrophages, and more advanced scaffold maturation. The presence of complex pore networks and high hydrodynamic resistance hinders the rapid formation of provisional biological scaffolds in porous structures. This hinders immune cell migration and vascularization efficiency, resulting in reduced tissue ingrowth. Conversely, the defined geometry, continuous luminal space, low hydrodynamic resistance, and efficient axial transport capacity of hollow channels facilitate the rapid delivery of fibrinogen and platelets to deep regions. These structural advantages trigger a series of biological reactions that rapidly form high‐quality, macrophage‐rich NETs‐fibrin biological scaffolds. Even the sparse structure deep within the channel was highly effective at guiding the rapid growth of new blood vessels, highlighting a key strength of the ability of NETs‐fibrin macrophage axis to provide vital initial support and facilitate the growth of new, functional blood vessels. While they are indispensable, NETs alone are insufficient to drive rapid vascular guidance independently. They play a crucial role in forming the NETs‐fibrin scaffold and recruiting macrophages. This enables the formation of the true orchestrator: the macrophage‐enriched biological scaffold that directs rapid vascular ingrowth in the channel.

The blood clots in question are fibrin‐crosslinked hydrogels containing platelets, neutrophils, monocytes/macrophages, and active growth factors.^[^ ^43^ ^]^ They play vital roles in revascularization and bone regeneration, and they have well‐established clinical applications in dental pulp and guided bone regeneration.^[^ ^44^, ^45^, ^46^, ^47^ ^]^ The cross‐linked fibrin network provides essential extracellular matrix support for cell adhesion and proliferation. Incorporated growth factors recruit immune cells and endogenous mesenchymal stem cells, thereby modulating immune responses and shaping the microenvironment. These properties make the clots ideal substitutes for channel‐formed provisional matrices. Despite their advantages, which include low cost, clinical accessibility, simple preparation, and high biocompatibility, limitations imposed by their inherent structural instability and poor mechanical strength restrict their standalone applications. These limitations can be overcome by incorporating mechanically robust, channeled SF scaffolds with host‐derived blood clots.

Preloading blood clots onto channeled SF scaffolds enables uniform the perfusion of activated platelets, immune cells, and fibrin networks into the deep core regions of the scaffold ex vivo. This process effectively recapitulates the 24–48 h in vivo process of immune‐complex matrix formation. This strategy significantly accelerates the vascularization and tissue integration of channel‐arrayed scaffolds. In conjunction with locally released BMP‐2, the blood clot preloading strategy enhances the osteogenic–angiogenic coupling effect. This approach increases bone volume and improves vertical bone height, demonstrating robust efficacy in promoting comprehensive bone regeneration. Using autologous blood for blood clot priming during surgical procedures has multiple advantages and demonstrates strong potential for clinical translation and application.

In this study, we systematically elucidated the fundamental mechanisms by which hollow‐channel structures accelerate vascularization through immunomodulation. This process relies on the efficiently guiding of the perfusion of macrophage‐rich, NET‐fibrin interwoven biological scaffolds within the channel structure. Inspired by this mechanism, we developed a biomimetic blood clot preloading strategy that works together with the channel structure to improve the constructs' overall vascularization. Along with localized BMP‐2 release, this innovative approach enhances the angiogenic–osteogenic coupling effect, significantly improving bone regeneration. These findings demonstrate significant translational potential, offering a clinically feasible solution for enhanced vascularized bone regeneration, which is a critical unmet need in reconstructive surgery and orthopedics.

## 4 Experimental Section

### Study Design

All in vitro and in vivo experiments were independently replicated at least three times. The data were analyzed using the appropriate statistical methods. For the in vivo studies, the rats were randomly assigned to the experimental groups. Surgical procedures and sample analyses adhered to principles of blinding and consistency. Sample sizes (n) are detailed in figure legends. The statistical methods are described in the “Statistical Analysis” section.

### Scaffolds Preparation

Briefly, sliced silk cocoons were boiled in 0.02 m sodium carbonate solution for 30 min and then rinsed with distilled water to ensure thorough purification. The purified SF was then dissolved in a 9.3 m lithium bromide (LiBr). The solution was then dialyzed (MWCO: 3500) and freeze‐dried to obtain the purified SF.

For freeze‐drying, the SF solution was mixed with horseradish peroxidase (HRP, Yeasen Biotechnology, Shanghai, China) and hydrogen peroxide (H^2^O^2^, Yeasen Biotechnology, Shanghai, China) at a ratio of 20 mg SF:10 U HRP:1.65 mm H_2_O_2_. The mixture was then transferred to a silicone mold containing an array of stainless steel rods with diameters of 300, 500, 700 and 900  µm. It was crosslinked at 37 °C, frozen at ‐80 °C for 24 h, and then lyophilized for 72 h.

For the particle‐leaching method, sucrose particles (200–300 µm) were compacted into silicone molds with minimal water. The cubic was then dried and demolded. The templates were then immersed in a solution of 20 wt% SF in hexafluoroisopropanol (HFIP). Then, they were transferred to methanol for 4 h, rinsed with running water, and soaked in deionized water for over 48 h to remove the HFIP and sucrose. Then, channels (500 µm in diameter) were drilled into the scaffolds. The scaffold morphology was then imaged with a macrozoom microscope (Olympus Corporation, Center Valley, PA).

### Animals

Male Sprague‐Dawley (SD) rats (8‐week‐old) and C57BL/6 mouse (8‐week‐old) were obtained from the Animal Laboratory Center of Shanghai Ninth People's Hospital, affiliated with Shanghai Jiao Tong University. Subcutaneous transplantation and calvarial bone augmentation models were established. All procedures were approved by the Institute for Laboratory Animal Research at the Ninth People's Hospital, affiliated with Shanghai Jiao Tong University, School of Medicine (SH9H‐2020‐A227‐1).

Antibody‐mediated neutrophil depletion: For neutrophil depletion experiments, mice were intraperitoneal injected with 200 µg per mouse of a neutrophil‐depleting antibody (anti‐Ly6G; BioLegend) 24 h before subcutaneous transplantation. Subsequently, these mice were further injected intraperitoneally with another dose of the antibody every 3 days.

### Subcutaneous Implantation Model

Under isoflurane anesthesia, a longitudinal incision was made in the dorsal region to expose the subcutaneous tissue. SF scaffolds (with or without channels) were implanted and sutured. Samples were harvested at predetermined time points for analysis. For the Adrenaline group, the channeled scaffolds were preinfiltrated with adrenaline (10 mg mL^−1^). For the Blood group, channeled scaffolds were preimmersed in fresh rat tail vein blood and allowed to clot for 1 h.

### Rat Calvarial Bone Augmentation Model

SF scaffolds were prepared, either without channels or with an array of 16 channels distributed across an area measuring 8 mm × 8 mm × 2 mm. BMP‐2 (0.15 mg mL^−1^) in sterile PBS was absorbed onto the scaffolds, followed by freezing (‐80 °C, 24 h) and lyophilization (24 h). For the Blood group, BMP‐2‐loaded channeled scaffolds were coated with fresh rat tail vein blood and allowed to clot for 1 h. Control groups consisted of: 1) BMP‐2‐loaded nonchanneled scaffolds (Control group) and 2) BMP‐2‐loaded channeled scaffolds (Channel group). Under isoflurane anesthesia, a midline scalp incision was made to expose the calvaria, and two 1 mm diameter burr holes were drilled. The scaffolds were positioned on the calvarial surface and sutured. After 4 weeks, the samples were harvested, fixed in 4% paraformaldehyde (PFA) for 24 h, and analyzed using micro‐CT (Bruker, MA). 3D reconstruction and quantification of new bone volume/height were performed using Avizo (v2020.1).

### Microfil Perfusion

Cylindrical scaffolds (10 mm diameter × 2 mm height, with or without channels) were implanted subcutaneously for 2 weeks. Following a previously described protocol, rats were perfused with Microfil (Flow Tech, MA).^[^ ^48^ ^]^ Briefly, under isoflurane anesthesia, the abdominal aorta was exposed and cannulated. Each rat was perfused with 20 mL of Microfil. The rats were then maintained at 4 °C for 1 h to permit Microfil polymerization. Samples were harvested, and fixed. The newly formed blood vessels were imaged and analyzed via micro‐CT (Bruker, MA).

### Histological Observation

At predetermined time points, samples were harvested and fixed in 4% PFA before being embedded in paraffin. Serial sections (4–6 µm) were prepared for histological analysis, which included H&E staining, Masson's trichrome staining, and Gram staining (Solarbio Science and Technology, Beijing, China). Imaging was performed using either a microscope (Nikon, Tokyo, Japan) or a Pannoramic Digital Slide Scanner (3DHISTECH, Budapest, Hungary). The thickness of collagen deposition within pore/channel structures was quantified and analyzed using ImageJ software.

### Immunohistochemistry

For immunofluorescence staining, deparaffinized sections were subjected to antigen retrieval in citrate buffer (pH 6.0, 30 min). The sections were then incubated overnight at 4 °C with the following primary antibodies: anti‐CD31 (1:500; Servicebio, Wuhan, China), anti‐α‐SMA (1:500; Servicebio, Wuhan, China), anti‐fibrinogen (1:500; Servicebio, Wuhan, China), anti‐MPO (1:500; Servicebio, Wuhan, China), anti‐CD68 (1:500; Servicebio, Wuhan, China), anti‐GPIIIa (1:200; Servicebio, Wuhan, China), anti‐P‐selectin (1:200; Proteintech, China), anti‐PSGL‐1 (1:400; Servicebio, Wuhan, China), anti‐CitH3 (1:200; Abcam, Cambridge, UK), anti‐NE (1:200; ABclonal, Wuhan, China), anti‐CD177 (1:400; Servicebio, Wuhan, China), anti‐VEGF‐A (1:200; Servicebio, Wuhan, China), anti‐Ly6G (1:200; Servicebio, Wuhan, China)anti‐iNOS (:500; Servicebio, Wuhan, China) and anti‐Arg‐1 (1:500; Servicebio, Wuhan, China). The sections were then incubated with Alexa Fluor 594/647‐conjugated secondary antibodies (Yeasen Biotechnology, Shanghai, China) and counterstained with DAPI (Beyotime Biotechnology, Shanghai, China) or Sytox Green (KeyGEN Biotech, Nanjing, China). Imaging was performed using a confocal laser scanning microscope (Leica Microsystems) and analyzed using ImageJ. Images were acquired using a confocal laser scanning microscope (CLSM, Leica Microsystems, Wetzlar, Germany) and analyzed with ImageJ.

### Culture of Cell Lines

The HL‐60 and THP‐1 cell lines were purchased from Wuhan Servicebio Technology Co., Ltd. The cells were cultured in Roswell Park Memorial Institute (RPMI)‐1640 medium (BasalMedia Technologies, Shanghai, China) supplemented with 20% or 10% fetal bovine serum (FBS, Biological Industries, Kibbutz Beit HaEmek, Israel), respectively. The cells were cultured at 37 °C with 5% CO_2_. HL‐60 cells were passaged at 1–2 × 10^6^ cells mL^−1^ and differentiated with 1.3% DMSO for 5–6 days (dHL‐60). The THP‐1 cells were passaged at 0.5–1 × 10^6^ cells mL^−1^ and differentiated with 50 ng mL^−1^ of phorbol 12‐myristate 13‐acetate (PMA) (MedChemExpress, Shanghai, China) for 48 h.

### Cell Labeling

dHL‐60 nuclei were stained with 20 nm Syto Green (KeyGEN Biotech, Nanjing, China) for 20 min. The cell membranes of the dHL‐60 or THP‐1 cells were labeled with 10 µm DiI (Beyotime Biotechnology, Shanghai, China) for 20 min.

### Transwell Assay

The channeled scaffolds were implanted subcutaneously for 30 min. Then, they were explanted and subsequently placed in the lower chambers of the Transwell. dHL‐60 cells (2.5× 10^4^ cells per well) were seeded in the upper chambers (0.3 µm pores) for 4 h; the unmigrated cells remained in the upper chambers and were counted using a Countess II automatic cell counter. The percentage of migrated cells was then calculated.

### Under‐Agarose Migration Assay

Agarose was dissolved at a concentration of 1 wt% in HBSS and then solidified in a 60 mm dish. Then, eight sets of three 3‐mm‐diameter wells, spaced 3 mm apart, were punched. PPP and PRP were prepared by centrifugation, as previously described.^[^ ^49^ ^]^ After activation with calcium chloride (CaCl_2_), the PPP and PRP were added to the inner wells and HBSS was added to the outer wells as a control. The dish was then incubated for 4 h at 37 °C. Then, dHL‐60 cell suspensions (1×10^5^ cells per well) were added to the middle wells. The dish was subsequently incubated for an additional 24 h at 37 °C. The samples were fixed with 4% PFA, the nuclei stained with Sytox Green, and the samples imaged using a using a microscope (Nikon, Tokyo, Japan).

### Fibrinogen Deposition Assay

A mixture of Syto Green‐ and DiI‐labeled dHL‐60 cell suspensions (1×10^5^ cells mL^−1^) and an equal volume of a 4 g L^−1^ hFibrinogen solution was incubated for 15 min. Smears were then prepared. The samples were then fixed with 4% PFA and stained with fibrinogen immunofluorescence. Images were acquired using a CLSM (Leica Microsystems, Wetzlar, Germany). Then fluorescence intensity and colocalization analysis of DiI and fibrinogen were performed using ImageJ software.

### Fibrinogen Coating

For plate coating, the wells were incubated with a solution of hFibrinogen (4 g L^−1^) or hSA (40 g L^−1^) at 37 °C for 4 h. This was followed by washing with PBS and air‐drying. For scaffold coating, the scaffolds were immersed in an hFibrinogen solution (4 g L^−1^) at 37 °C for 4 h. Then they were thoroughly washed with PBS and the excess liquid was removed prior to subsequent assays.

### Neutrophils Adhesion Assay

A suspension of Syto Green‐labeled dHL‐60 cells (1 × 10^5^ cells mL^−1^) was added to hFibrinogen‐coated wells and incubated at 37 °C for 1 h. Nonadherent cells were removed by washing with PBS. Images were acquired using a CLSM (Leica Microsystems, Wetzlar, Germany). The nuclei stained with Syto Green were counted and statistically analyzed using ImageJ software.

### NETosis Assay

dHL‐60 cells (1 × 10^5^ cells mL^−1^) prelabeled with Syto Green and DiI were seeded onto hFibrinogen‐coated wells. Cells plated onto untreated wells were used as controls. The cells were then incubated at 37 °C for either 30 min or 1 h, and morphological observations were recorded using Morphological observations were recorded using a CLSM (Leica Microsystems, Wetzlar, Germany).

For immunofluorescence staining, dHL‐60 cells (1 × 10^5^ cells mL^−1^) were incubated in hFibrinogen‐coated wells at 37 °C for 1 h. The cells were then fixed with 4% PFA for 30 min and subjected to MPO and CitH3 immunofluorescence staining. Sytox Green was used for nuclear counterstaining. For 3D coculture system evaluation, Syto Green‐ and DiI‐prelabeled dHL‐60 cell suspensions (1 × 10^5^ cells mL^−1^) were added to hFibrinogen‐coated, channeled scaffolds first. After incubation at 37 °C for 1 h, the scaffolds were washed with PBS to remove nonadherent cells and fixed with 4% PFA for 30 min. Imaging was performed using a CLSM (Leica Microsystems, Wetzlar, Germany).

### Macrophage Chemotaxis Detection

A mixture of 150 µL of a 4 g L^−1^ hFibrinogen solution and an equal volume of a 1 × 10^5^ cells mL^−1^ dHL‐60 cell suspension was incubated in a glass vial at 37 °C to induce gelation.

The dHL‐60 cell nuclei were prelabeled with Syto Green, and the THP‐1 cell membranes were labeled with DiI. An equal volume of hFibrinogen solution (4 g L^−1^) and dHL‐60 cell suspension (1 × 10^5^ cells mL^−1^) were mixed to form a hydrogel. Then, THP‐1 cells (1 × 10^5^ cells mL^−1^) were seeded onto the gel. Images were taken at determined time points using a CLSM, (Leica Microsystems, Wetzlar, Germany)

For the Transwell assay, a mixture of hFibrinogen solution (4 g L^−1^) and a dHL‐60 cell suspension (1 × 10^5^ cells mL^−1^) was added to the lower chamber to form a hydrogel. The hydrogel was then carefully removed, leaving the adherent NETs cell layer intact. After washing thoroughly with PBS, the medium was replaced with RPMI‐1640 containing 10% FBS. Then, THP‐1 cells (2.5 × 10⁴ cells mL^−1^) were seeded in the upper chamber and cocultured for 24 h. The migrated cells were fixed with 4% PFA, stained with crystal violet and imaged (Nikon, Tokyo, Japan). ImageJ software was then used to quantify and statistically analyze the migrated cells.

### Statistical Analysis

All data are expressed as the mean ± standard deviation (SD). Statistical analyses were conducted using GraphPad Prism 10 software (GraphPad, USA). Significant differences were determined using a Student's *t*‐test or analysis of variance (ANOVA). A *p* value of < 0.05 was considered statistically significant. The symbols *, **, ***, and **** represent *p* values of < 0.05, < 0.01, < 0.001, and < 0.0001, respectively. n.s. indicates not significant.

## Conflict of Interest

The authors declare no conflict of interest.

## Supporting information



Supporting Information

## Data Availability

The data that support the findings of this study are available from the corresponding author upon reasonable request.
