# Supplementary material for: Elevator‐Like Hollow Channels in Porous Scaffolds Accelerate Vascularized Bone Regeneration via NETs‐Fibrin‐Mediated Macrophage Recruitment
Source: Adv Sci (Weinh). 2025 Dec 5;13(9):e15693. doi: 10.1002/advs.202515693 (PMC12904018; doi:10.1002/advs.202515693)
Supplement: Supplementary file 1 — Supporting Information [file ADVS-13-e15693-s001.docx]

Supporting Information

Elevator-like Hollow Channels in Porous Scaffolds Accelerate Vascularized Bone Regeneration via NETs-fibrin-mediated Macrophage Recruitment

Guifang Wang, Rongpu Liu, Huijing Ma, Shuhan Duan, Guangzheng Yang, LingXi Meng, Yuqin Qiao, Dongqiang Song*, Wenjie Zhang*

Supplementary Figures


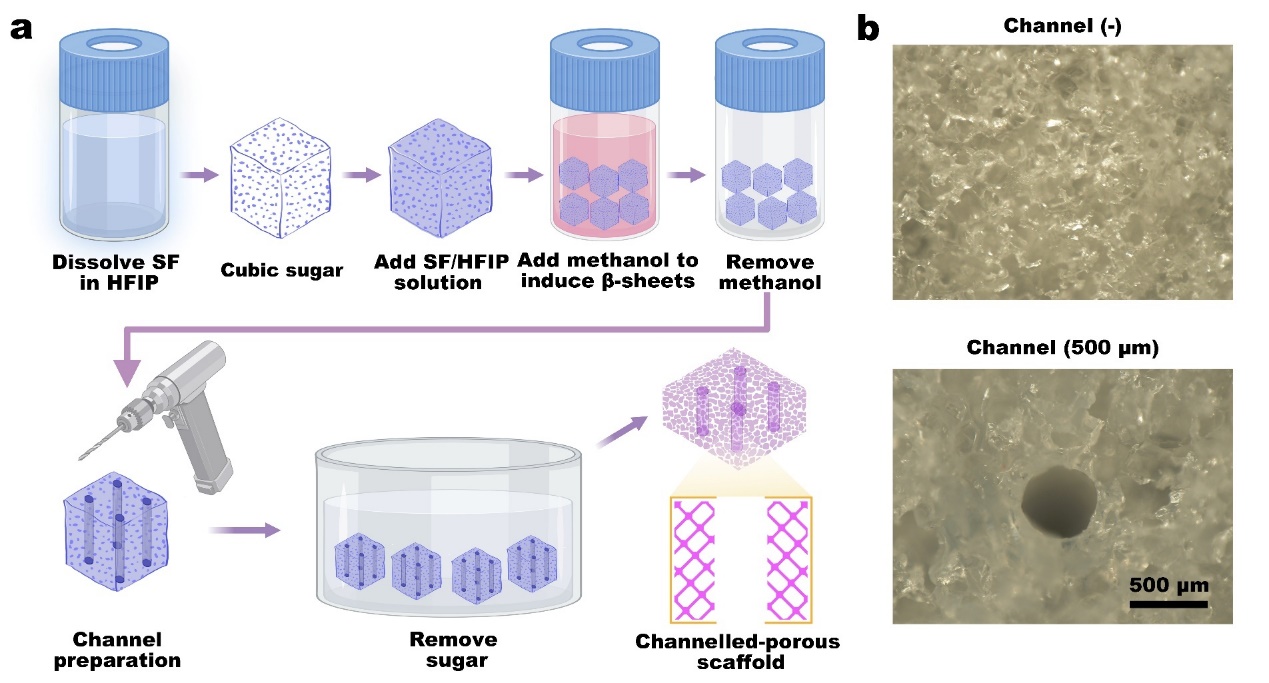


**Figure S1.** Fabrication of channeled scaffolds. (a) Schematic illustration of the particle-leaching method for preparing porous scaffolds with hollow channels. A 20 wt.% silk fibroin (SF)/hexafluoroisopropanol (HFIP) solution was prepared by dissolving SF in HFIP. This solution fully infiltrated sucrose particle templates (200-300 µm). The construct was then transferred to methanol for complete crosslinking. Hollow channels (500 μm in diameter) were created using a drilling process. Rinsing thoroughly in running water removes residual HFIP and sucrose particles, yielding porous SF scaffolds (pore size: 200-300 μm) with well-defined 500-μm channels. (b) Macro-morphology of scaffolds with and without channels.


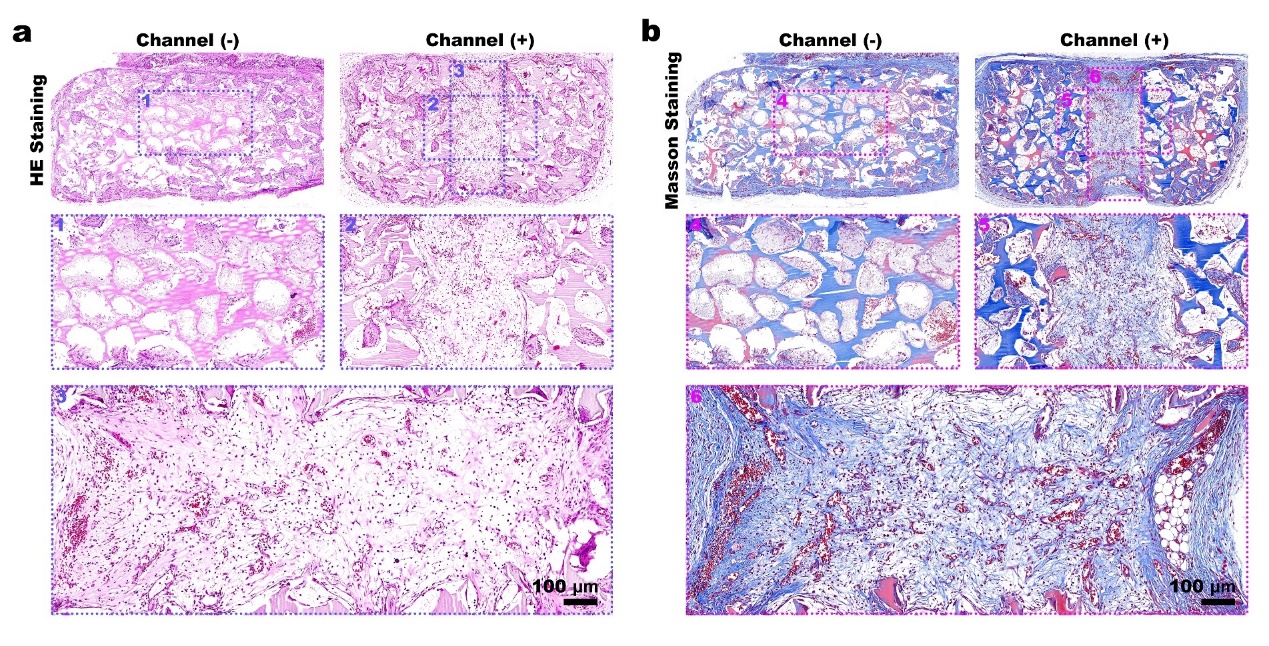


**Figure S2.** Histological observation of scaffolds with or without channels after 14 days of subcutaneous implantation in rats. (a) Hematoxylin and eosin (H&E) staining. (b) Masson's trichrome staining.


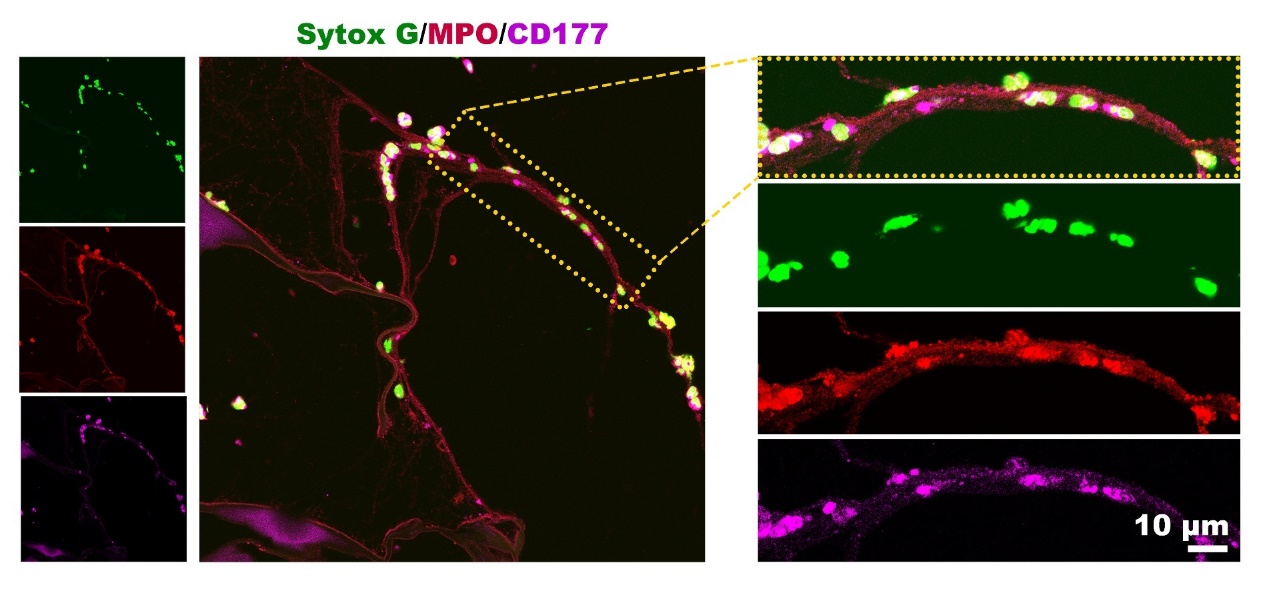


**Figure S3.** Immunofluorescence co-staining was conducted for CD177 and MPO at 4 h after subcutaneous implantation in rats, with the nuclei counterstained using Sytox Green.


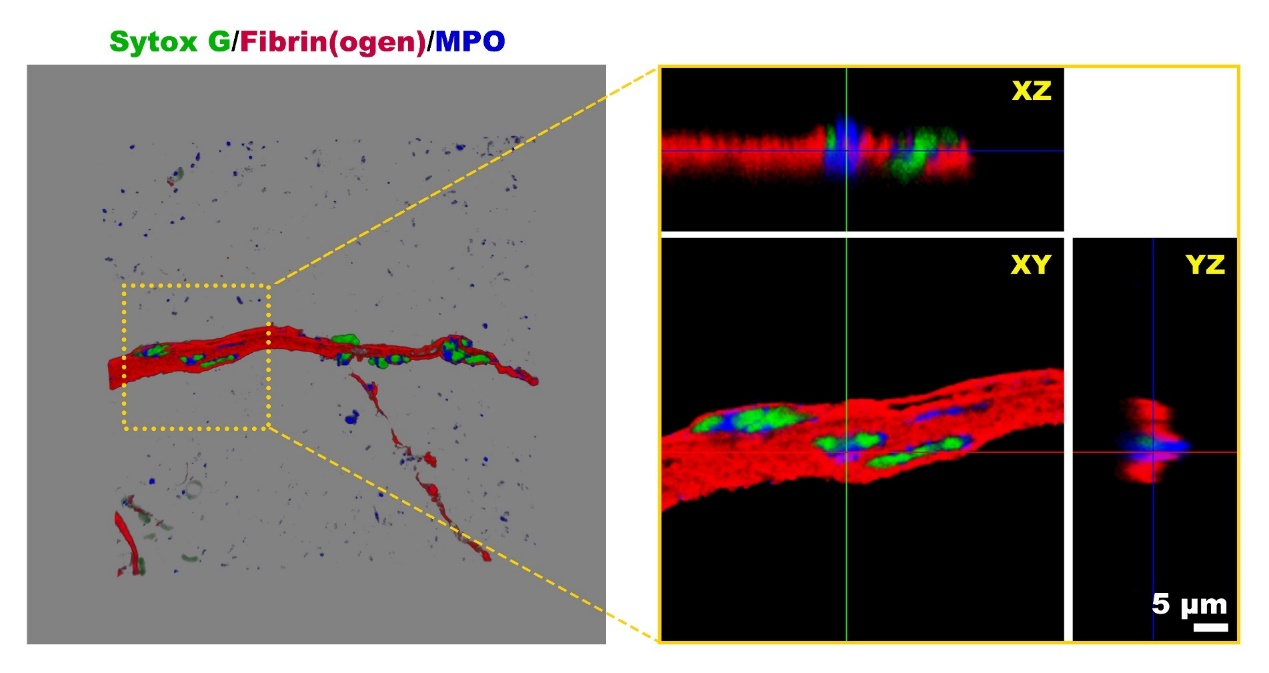


**Figure S4.** Immunofluorescence co-staining of MPO and Fibrinogen was conducted at 4 h after subcutaneous implantation in rats, with Sytox Green nuclear counterstain, showing three-dimensional reconstruction and representative orthogonal sections from confocal microscopy.


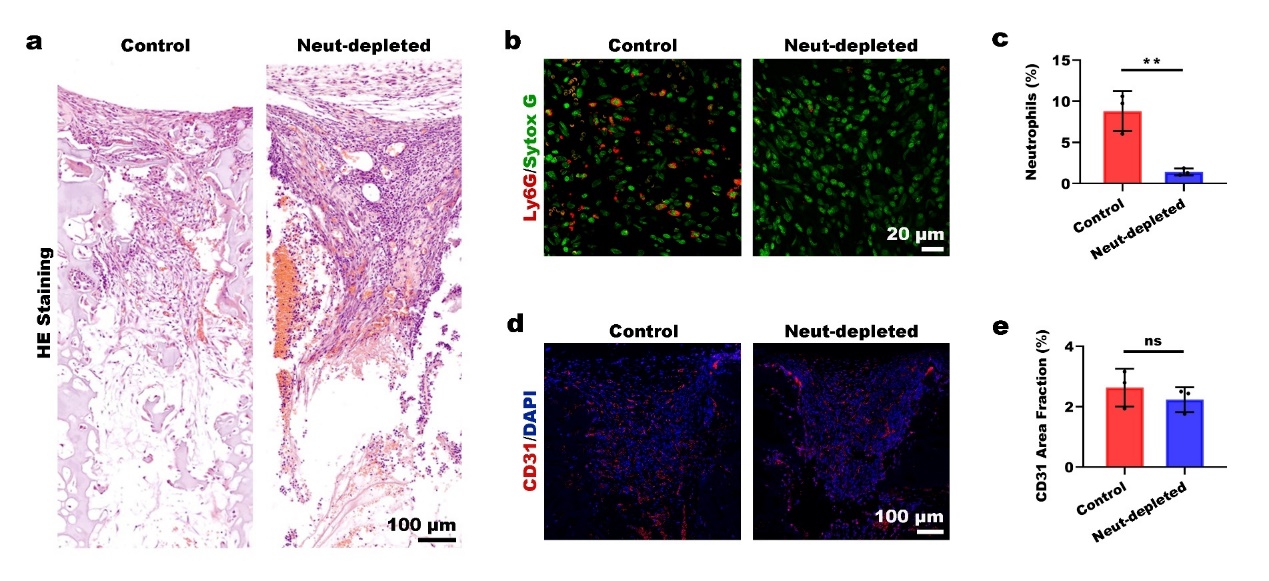


**Figure S5**. Neutrophil depletion experiments. After 14 days of subcutaneous implantation in rats, (**a**) H&E staining of the control and neutrophil-depleted groups was performed. (**b**) Immunofluorescence staining of Ly6G was conducted, with Sytox Green nuclear counterstain. (**c**) Statistical analysis of Ly6G^+^ neutrophil ratio was conducted (n=3 biological replicates). (**d**) Immunofluorescence staining of CD31 was conducted, with DAPI nuclear counterstain. (**e**) Quantification of CD31⁺ area in the control and neutrophil-depleted groups (n=3 biological replicates). Statistical analyses were carried out using one-way ANOVA with Tukey’s post hoc correction for (**c**) and (**e**). Significance levels: *P<0.05, **P<0.01, ***P<0.001, and ****P<0.0001.


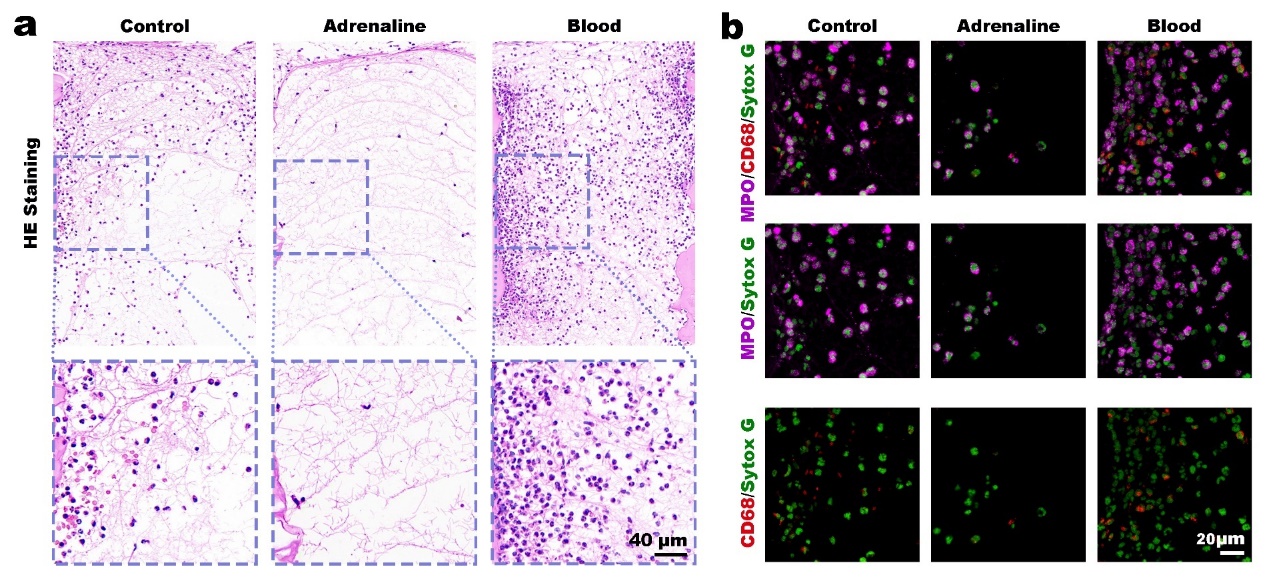


**Figure S6**. Preloaded blood clot enhances channel guided neutrophil and macrophage infiltration after 2 days of subcutaneous implantation in rats. (a) H&E staining. (b) Immunofluorescence co-staining was conducted for MPO and CD68, with the nuclei counterstained using Sytox Green.
